# SLAF-based high-density genetic map construction and QTL mapping for major economic traits in sea urchin *Strongylocentrotus intermedius*

**DOI:** 10.1038/s41598-017-18768-y

**Published:** 2018-01-16

**Authors:** Yaqing Chang, Jun Ding, Yuhui Xu, Dan Li, Weijie Zhang, Lei Li, Jian Song

**Affiliations:** 1 Dalian Ocean University, Key Laboratory of Mariculture & Stock Enhancement in North China’s Sea, Ministry of Agriculture, Dalian Liaoning, 116023 China; 2 Biomarker technology Corporation, Beijing, 101300 China

## Abstract

Sea urchin (*Strongylocentrotus intermedius*) has long been a model species for developmental and evolutionary research, but only a few studies have focused on gene mapping. Here, we reported a high-density genetic map containing 4,387 polymorphism specific-length amplified fragment (SLAF) markers spanning 21 linkage groups (LG) for sea urchin. Based on this genetic map and phenotyping data for eight economic traits, 33 potentially significant QTLs were detected on ten different LGs with explanations ranging from 9.90% to 46.30%, partly including 10 QTLs for test diameter, six QTLs for body weight and eight QTLs for Aristotle’s lantern weight. Moreover, we found a QTL enrichment LG, LG15, gathering QTLs for test diameter, body weight, gonad weight, light orange-yellow color difference (≥*E*_1_) and light yellow color difference (≥*E*_2_). Among all QTLs, we genotyped four QTLs for test diameter, Aristotle’s lantern weight and body weight using High Resolution Melting (HRM) technology. Finally, we used the verified SNP marker (detected using SLAF sequencing) to explore their marker-assisted selection (MAS) breeding application potential and found that SNP-29 associated tightly with body weight and that heterozygous genotype was a dominant genotype, indicating that SNP-29 was a promising marker for MAS.

## Introduction

Sea urchin (*Strongylocentrotus intermedius*) is native to the eastern Pacific Ocean, mainly in the northern Sea of Japan and the Russian far coast. It was transplanted to China in 1989 and has since become one of the three most important cultured sea urchin species in China, especially in Liaoning and Shandong Provinces, in which the production of sea urchin reached 5066 tons in 2015^1^.

Sea urchin has long been a model species for developmental and evolutionary studies. The release of the sea urchin *Strongylocentrotus purpuratus* genome sequence in 2006 has greatly facilitated genetic and genomic research^2^. However, it is necessary to comprehensively understand the genomic and genetic characteristics of sea urchin and provide support for the cultivation of breeding. A genetic map, especially a high-density genetic map, provides an important foundation for QTL mapping, and some underlying molecular markers can be used for marker-assisted breeding programs to satisfy demand^3^. Recently, high-density genetic linkage maps have been constructed for many aquaculture species, including sea cucumber^4^, pacific white shrimp^5^, *Hyriopsis cumingii*^6^, *Haliotis diversicolor*^7^, *Odontobutis potamophila*^8^, and *cophthalmus maximus*^9^. To our knowledge, only two genetic maps for sea urchin have been constructed^10,11^. A high-density linkage map was constructed in 2015, and based on this genetic map, several economic-related QTLs were mapped. To date, only a few QTLs mapping for *S*. *intermedius* have been identified based on AFLP or SNP markers. Few studies with QTL verifying and informative markers development for MAS breeding have been published.

In this study, we employed a recently developed SLAF-seq approach to achieve rapid discovery of SNP markers for sea urchin. Using these newly developed markers, a high–density genetic map was constructed, and the QTLs for major economic traits were mapped. HRM analysis was applied to verify the genotyping results for some QTLs by SLAF-seq and screen potential functional SNP markers for MAS breeding.

## Results

### High-throughput SLAF sequencing and genotyping

*Rsa*I and *Hae*III were chosen for genome DNA digestion according to an *in silico* restriction enzyme digestion prediction based on the *Strongylocentrotus purpuratus* genome (http://www.ncbi.nlm.nih.gov/genome/?term=strongylocentrotus%20purpuratus). A rice restriction enzyme digestion control trial was conducted to monitor the progress of the sea urchin SLAF library construction indirectly. Based on the control, the ratio of pair-end mapping reads was 79.66% and the percentage of digestion was 90.83%, indicating that a normal SLAF library was constructed.

A total of 533.05 M reads with Q30 of 89.03% and GC content of 37.39% was generated from one lane of the Illumina sequencing flowcell. The total reads of the two parents were 13,355,278 and 9,550,588; 2,629,599 reads were generated on average in the F_1_ offspring (Table 1).

**Table 1:** Statistics for sequenced data.

Sample ID	Total reads	Q30 percentage (%)	GC percentage (%)
Male	13,355,278	87.72	36.45
Female	9,550,588	87.44	37.01
Offspring	2,629,599	89.05	37.39
Control (Rice)	239,728	84.99	41.61

After bioinformatics analysis, data for 150 individuals was used to develop SLAF-tags: 249,076 SLAF tags were developed in the male parent and 279,416 in the female parent, with an average depth of 32.62-fold and 20.53-fold, respectively. For the F_1_ mapping population, 190,361 SLAF-tags were generated with an average depth of 8.80-fold (Supplementary Table 1). For F_1_ individuals, the marker depth ranged from 1.21-fold to 19.13-fold, while the SLAF-tags were between 18,305 and 235,095 (Supplementary Fig. 1).

**Figure 1:**
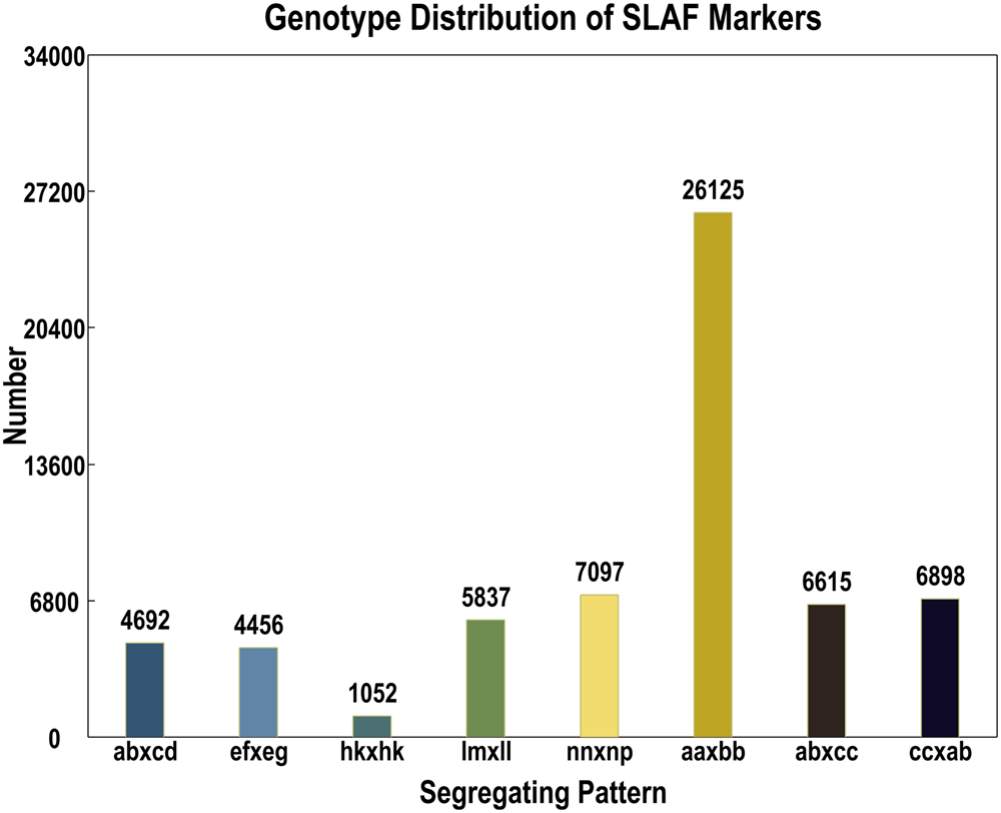
Segregation pattern of polymorphic SLAF markers.

The developed SLAF-tags can be divided into three groups: polymorphic, non-polymorphic and repetitive. A total of 197,375 polymorphic SLAF markers were generated and used for the next encoding progress, and 62,772 polymorphic SLAF markers were successfully classified into eight segregation patterns (Fig. 1). Patterns except aa × bb were selected for later linkage map construction suitable for the F_1_ population, and 36,647 SLAF markers were left for map construction. The effective polymorphism rate was 7.99%.

### Genetic linkage map construction

After four quality control steps, 4,678 reserved polymorphic SLAF markers were loaded into HighMap software to construct a genetic map^12^. Two linkage maps (male map and female map) and an integrated map were constructed, including 21 linkage groups (LGs). The visual graph of the sex-average genetic map is shown in Supplementary Fig. 2. The statistics results are exhibited in Table 2. In total, 4,387 SLAFs were mapped onto the integrated genetic map. The average depth of the SLAFs on the map was 120.97-fold in the male parent and 101.33-fold in the female parent; it was 26.64-fold in the F_1_ population. The male map spanned 3,116.10 cM, with 983 markers and an average inter-marker spacing of 3.24 cM. The length of the female map was 3,537.39 cM, with 3,465 markers and an average inter-marker spacing of 1.03 cM. The length of the integrated map was 1,907.21 cM, and the average distance between adjacent markers was 0.44 cM. For the integrated map, the number of markers ranged from 81 to 498 for each LG, with an average of 209 markers per LG. The sizes of the individual LGs varied from 71.80 to 119.14 cM, with average inter-marker distances of 0.21–1.18 cM. LG20 was not only the longest but also the densest group, with 498 loci spanning 119.14 cM. LG18 was the shortest, with 177 loci spanning 71.8 cM; LG4 contained the fewest markers (81 markers).

**Table 2:** Summary of three linkage maps of sea urchin.

LG ID	Average map				Male map				Female map			
	Total Marker	Total Distance (cM)	Average Distance (cM)	Max Gap (cM)	Total Marker	Total Distance (cM)	Average Distance (cM)	Max Gap (cM)	Total Marker	Total Distance (cM)	Average Distance (cM)	Max Gap (cM)
LG1	247	93.76	0.38	14.72	104	105.36	1.02	14.73	146	125.30	0.86	9.52
LG2	116	88.44	0.77	10.92	19	120.91	6.72	31.40	99	184	1.88	24.44
LG3	207	91.92	0.45	8.95	26	177.88	7.12	38.11	183	146.33	0.80	11.16
LG4	81	94.40	1.18	8.19	7	99.96	16.66	25.54	76	76	1.01	9.52
LG5	273	99.76	0.37	9.47	75	90.29	1.22	6.64	199	379.86	1.92	19.28
LG6	152	100.08	0.66	15.86	36	279.45	7.98	42.59	119	140.07	1.19	5.64
LG7	135	72.25	0.54	8.75	35	193.95	5.70	31.43	102	151.09	1.50	23.37
LG8	198	88.84	0.45	7.47	39	88.87	2.34	9.66	161	130.27	0.81	9.14
LG9	189	78.93	0.42	16.09	44	248.54	5.78	47.51	148	177.98	1.21	14.61
LG10	258	93.46	0.36	13.22	88	93.46	1.07	14.28	171	123.44	0.73	7.16
LG11	253	93.55	0.37	12.50	54	97.44	1.84	13.79	201	182.79	0.91	9.52
LG12	193	76.58	0.40	12.79	44	83.13	1.93	9.84	152	140.51	0.93	7.16
LG13	268	106.47	0.40	9.32	72	90.51	1.27	12.68	199	137.32	0.69	11.16
LG14	360	84.86	0.24	8.79	82	444.91	5.49	31.43	285	53.53	0.19	4.20
LG15	178	109.49	0.62	11.83	37	272.24	7.56	36.7	145	111.93	0.78	5.64
LG16	127	75.86	0.6	4.52	12	15.04	1.37	3.45	119	369.47	3.13	24.44
LG17	189	91.73	0.49	14.45	35	91.73	2.70	14.45	155	119.45	0.78	8.72
LG18	177	71.80	0.41	7.16	49	70.52	1.47	13.04	132	90.72	0.69	13.72
LG19	163	75.30	0.46	6.51	12	79.65	7.24	25.54	153	184.9	1.22	26.66
LG20	498	119.14	0.24	5.82	89	94.74	1.08	8.93	417	400.33	0.96	19.28
LG21	125	100.59	0.81	14.57	24	277.52	12.07	39.42	103	112.10	1.10	11.16
Total	4,387	1,907.21	0.44	16.09	983	3,116.10	3.24	47.51	3,465	3,537.39	1.03	26.66

Segregation distortion (SD) is a common natural phenomenon and may reflect early life history of mortality in crosses of bivalves^13^. So we checked the segregation degree of all the SLAF markers by Chi-square test in this sex-average genetic map and found that 85 out of 4,387 SLAF markers showed extremely SD (*P* < 0.01, Supplementary Table 2). The extremely distorted SLAF markers were mainly located on LG16, LG19 and no distorted marker was found on LG3, LG9 and LG17 according to a genome-wide scan in Supplementary Fig. 3.

### Genetic map evaluation

The quality of this genetic map was evaluated by heat maps and haplotype maps, as done in *Salvia miltiorrhiza*^14^, mango^15^ species, which directly reflected the recombination relationships among markers in the 21 LGs. Heat maps were constructed by using pair-wise recombination scores for the 4,378 SLAF markers (Supplementary Fig. 4), indicating that SLAF markers’ ordination and the genetic distance of adjacent markers in most LGs were accurate.

Haplotype map was used to reflect the crossover evens. The double crossover rates of all LGs ranged from 0 to 0.83% (Supplementary Table 3; Supplementary Fig. 5), suggesting that this high-density genetic map was suitable for later genetic analysis.

### Phenotypic variation analysis

**Figure 2:**
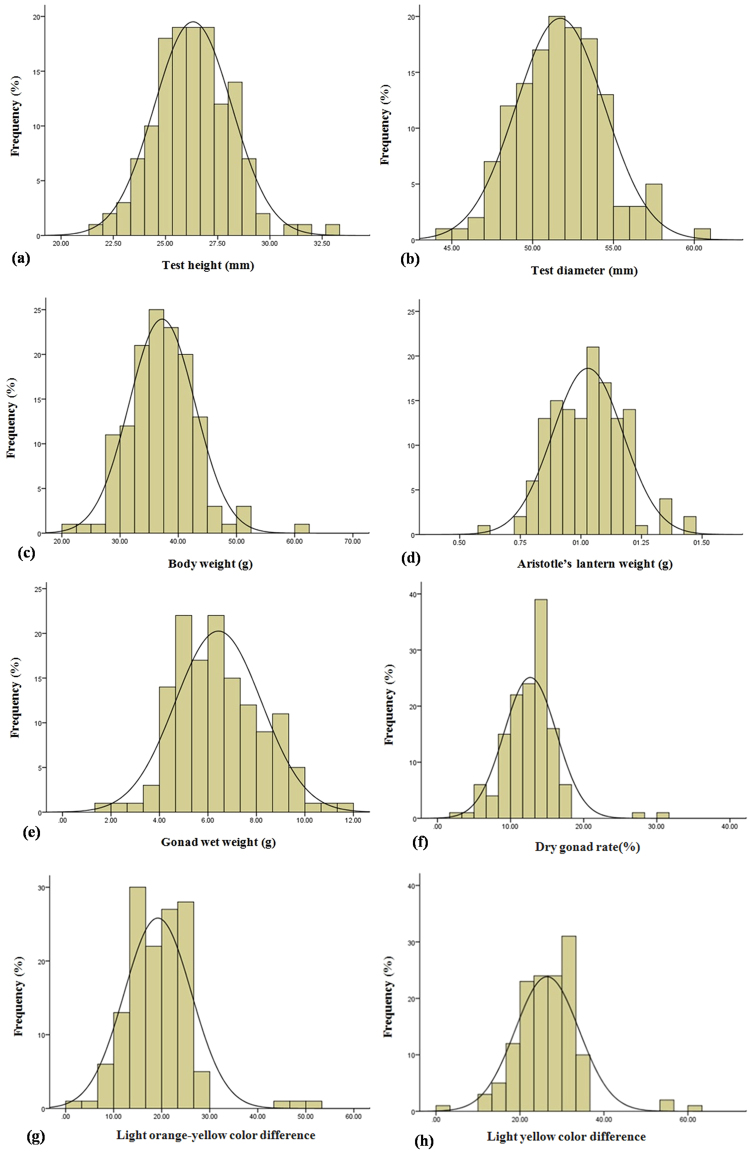
Frequency distributions of eight traits.

Table 3 shows statistics analysis of economic traits in QTL mapping population containing 150 individuals. In total, we analyzed eight population traits. The mean test height, test diameter, body weight and Aristotle’s lantern weight, gonad wet weight, dry gonad rate, light orange-yellow color difference and light yellow color difference were 26.32 mm, 51.71 mm, 37.22 g, 13.05%, 1.03 g, 6.43 g, 12.69%, 19.19 and 26.16, respectively. The coefficient of variation (CV) of all traits ranged from 5.30% to 36.48%, and the light orange-yellow color difference was greatest, while the test diameter had the lowest value. The absolute values of skewness for all traits except light orange-yellow color difference were less than 1, which suggested that those traits were distributed normally. Kurtosis fluctuated aggressively on the whole rather than skewness. Meanwhile, the *P*-value of the normality test shared the same results and was much larger than the threshold (0.05). The frequency distributions of eight traits for the population were also analyzed (Fig. 2), and we also found that the eight traits exhibited normal distribution suitable for QTL detection.

**Table 3:** Statistics analysis of phenotypic trait of QTL mapping population.

Traits	Mean ± SD	Skewness	Kurtosis	CV (%)	*P* value
Test height (mm)	26.32 ± 1.85	0.39	0.99	7.03	0.99
Test diameter (mm)	51.71 ± 2.74	0.16	0.20	5.30	0.99
Body weight (g)	37.22 ± 5.66	0.53	1.81	15.21	0.85
Aristotle’s lantern weight (g)	1.03 ± 0.15	0.18	0.24	14.56	0.95
Gonad wet weight (g)	6.43 ± 1.79	0.33	0.04	27.84	0.72
Dry gonad rate (%)	12.69 ± 3.60	0.79	5.19	35.00	0.20
Light orange-yellow color difference (△*E*_1_)	19.19 ± 7.00	1.33	5.64	36.48	0.15
Light yellow color difference (△*E*_2_)	26.46 ± 7.59	0.79	4.84	28.68	0.09

*P* > 0.05 indicates a normal distribution.

### QTL mapping analysis

**Figure 3:**
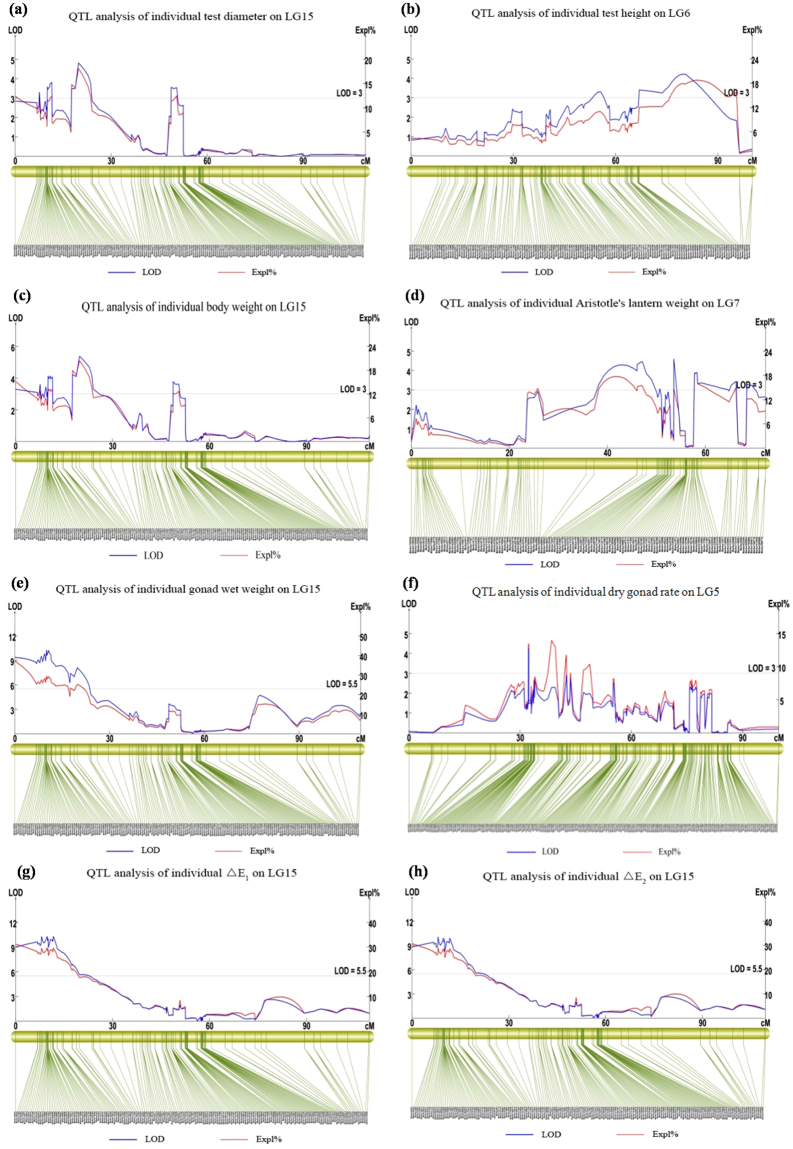
QTL analysis of different traits in different LGs. Blue line and red line represent LOD score and explained phenotypic variation of QTL, respectively.

Using MapQTL under the interval mapping model, a total of 33 QTLs were detected, interspersed on 13 different LGs (LG1, LG2, LG3, LG4, LG5, LG6, LG7, LG10, LG12, LG14, LG15, LG17, and LG20). Detailed information was provided in Table 4 and Fig. 3. Multiple QTLs on different LGs were mapped for traits except for test height, dry gonad rate, *ΔE*1 and *ΔE*2. The only QTL for test height was detected in LG6, explaining 17.80% of its phenotypic variation. On LG4, a QTL for dry gonad rate near Marker 63107 explained up to 46.30% of phenotypic variation. Aristotle’s lantern weight QTL was located on five disparate LGs (LG1, LG2, LG3, LG7, and LG17), and the maximum explanation reached 18.60%. Test diameter QTL, however, was located on LG6, LG7, LG10, LG12, LG15 and LG20, with a maximum explanation of 18.1%, indicating a more complex genetic mechanism than for other traits. Although QTL for test diameter was located on 6 LGs, LG15 encompassed almost half of the QTLs. Similarly, QTLs for body weight and gonad wet weight, the most important commercial characteristics, were also found on LG15 and explained 20.4% and 37.2% phenotypic variation, respectively, and the LOD was 10.27 for gonad wet weight QTL. In addition, *ΔE*1 and *ΔE*2 QTL were on LG15 with 30% explanation, approximately, and LOD above 10 for *ΔE*1 and *ΔE*2, respectively. All QTLs indicated that LG15 played a very important role for yield and quality formation and that more attention should be paid to this linkage group in future research.

**Table 4:** QTL mapping information for economic traits in sea urchin.

Traits	Linkage groups	Position (cM)	Peak markers	LOD	Explanation (%)
Test diameter	LG6	29.82	Marker27975	3.10	10.20
Test diameter	LG6	55.71	Marker53227	3.02	9.90
Test diameter	LG7	51.85	Marker423802	3.27	10.90
Test diameter	LG10	41.84	Marker209362	3.10	10.10
Test diameter	LG12	37.57	Marker42649	3.52	12.20
Test diameter	LG15	19.78	Marker212278	4.81	18.10
Test diameter	LG15	11.55	Marker30866	3.82	12.50
Test diameter	LG15	48.74	Marker212773	3.56	11.40
Test diameter	LG15	18.78	Marker270696	3.49	14.20
Test diameter	LG20	77.18	Marker19255	3.49	11.20
Test height	LG6	80.47	Marker356502	4.21	17.80
Test height	LG6	66.92	Marker101079	3.40	12.00
Body weight	LG15	19.78	Marker212278	5.39	20.40
Body weight	LG15	10.07	Marker60849	4.15	13.40
Body weight	LG15	48.74	Marker212773	3.79	12.10
Body weight	LG15	7.59	Marker13442	3.62	12.80
Body weight	LG15	24.02	Marker1338	3.30	11.10
Body weight	LG20	79.70	Marker85134	4.98	15.50
Aristotle’s lantern weight	LG1	67.93	Marker81106	3.12	12.90
Aristotle’s lantern weight	LG2	54.18	Marker17945	3.14	11.00
Aristotle’s lantern weight	LG3	78.84	Marker59840	3.20	16.60
Aristotle’s lantern weight	LG7	53.57	Marker650662	4.60	14.70
Aristotle’s lantern weight	LG7	47.19	Marker13837	4.43	15.20
Aristotle’s lantern weight	LG7	58.29	Marker104973	3.90	18.60
Aristotle’s lantern weight	LG7	68.47	Marker649094	3.28	12.20
Aristotle’s lantern weight	LG17	50.24	Marker90893	3.28	10.60
Gonad wet weight	LG15	10.07	Marker60849	10.27	29.50
Gonad wet weight	LG15	0	Marker29645	9.37	37.20
Dry gonad rate	LG4	37.78	Marker63107	3.93	46.30
Dry gonad rate	LG5	32.34	Marker365114	4.26	13.50
Dry gonad rate	LG14	56.96	Marker21620	3.14	10.10
Light orange-yellow color difference (△*E*_*1*_)	LG15	11.69	Marker9192	10.31	29.60
Light yellow color difference (△*E*_*2*_)	LG15	8.04	Marker6879	10.06	29.10

### HRM analysis

HRM is a rapid, high-throughput and efficient method for variation identification, especially for SNP genotyping^16,17^. In this paper, 10 primers were developed from our prior QTL information. After a primary screen by the Light Scanner 96 system, primers of SNP-22 (marker17945), SNP-29 (marker19255) and SNP-37 (marker85700) responsible for Aristotle’s lantern weight, test diameter/body weight, and test diameter were kept for later HRM genotyping, respectively. More detailed information is shown in Supplementary Table 4. From Supplementary Table 4, all three SNPs associated with economic-related QTLs showed explanation greater than 10%.

Our HRM genotyping results for the three SNPs above are displayed in Supplementary Fig. 6. Different genotypes demonstrated different curves. The homozygous genotype was unimodal, while the heterozygous genotype was double-humped and the Tm value of G/C was higher than for A/T. All three SNP exerted polymorphism genotypes, suggesting that a genetic map that employed reliable SNP markers was constructed.

Ninety individuals from three additional families were genotyped through the three SNPs, but only SNP-29 showed promising functional molecular markers (Supplementary Table 5, Supplementary Fig. 7). Fifty-nine individuals were identified as homozygotes, and the rest were heterozygotes for SNP-29 (Table 5). There was a highly significant difference (*P*  < 0.01) between them, and the explanation fluctuated at approximately 40%, indicating that heterozygous SNP-29 was a dominant genotype and a promising marker for sea urchin molecular breeding.

**Table 5:** SNP-29 genotyping in three new populations.

Traits	Homozygous genotype	Heterozygous genotype	*SSt*	*SST*	*P* (%)
Sample No.	59	31	—	—	—
Test height (mm)	18.51 ± 4.47^A^	22.45 ± 2.38^B^	7.76	17.48	44.40
Test diameter (mm)	34.57 ± 10.34^A^	43.69 ± 6.17^B^	41.59	101.53	40.96
Body weight (g)	18.83 ± 14.14^A^	31.26 ± 10.82^B^	77.25	205.13	37.66

Capital letter A and B at top-right corner means highly significant differences detected between the two genotypes. (*P* < 0.01); (*P*) = *SS*_*t*_/*SS*_*T*_ × 100%.

## Discussion

NGS-based genotyping technology plays an increasingly important role in the identification of large numbers of molecular markers such as SNPs. Many SNP-based markers developed by reduced-representation sequencing or whole genome resequencing were used for high-density genetic map construction and QTL mapping^4,18–20^. In this study, a high-density genetic map for sea urchin was constructed using SLAF-seq technology, and many economic-related QTLs were detected. SLAF-seq technology was also successfully applied in other aquatic species^5,6,8,21^, indicating that SLAF-seq is an effective method for high-density genetic map construction. In addition, HRM was used to genotype some SNPs conferring faster growth, and an informative SNP marker, called SNP-29, has potential for MAS breeding.

Sea urchins have been model organisms for developmental biology and evolutionary studies, but little development of functional gene mining by genetic maps has been achieved. In 2006, Zhou *et al*. constructed an AFLP linkage map of sea urchin^10^. The female genetic map was composed of twenty-four linkage groups and contained 194 AFLP markers, covering a total length of 2988.3 cM with an average marker spacing of 17.1 cM. For the male genetic map, 199 AFLP markers were mapped in 23 linkage groups, covering a total length of 2614.8 cM with an average of 15.4 cM between markers. Our genetic map shows progress regardless of marker numbers or linkage group numbers. Nevertheless, the integration of SNP markers and AFLP markers may be necessary to obtain a consensus genetic map. The total genetic distance of the female map (3537.39 cM) was obviously longer than for the male map (3116.10 cM), similar to total marker numbers. This phenomenon was also discovered in other studies of different species such as sea urchin^11^, rainbow trout^22^, zebrafish^23^, fugu^24^, Japanese eel^25^ and *Hyriopsis cumingii*^6^, indicating fewer recombination events in male parents. Sex-specific recombination has been recognized for a long time, and several explanations exist. Bernstein^26^ thought that higher metabolic activity in female individuals and oxidative damage in eggs requires higher recombinational repair than male individuals. Trivers^27^ noted that although the actual combinations of male genes are important to their success, they will be selected, reducing rates of recombination (compared to females). But further investigations is needed whether this principle operates in aquatic animals.

Recently, a high-density genetic map in sea urchin was released by RAD-seq^11^. A total of 3080 and 1577 markers were mapped onto the female genetic map and male map, respectively. Based on this genetic map, a QTL controlling body size was identified on LG 5 spanning from 25.3 cM to 30.3 cM. Our study detected 33 QTLs located on 13 different LGs and only one dry gonad rate QTL located on LG5, implying a complicated genetic mechanism under economic trait formation. In addition, we found that a QTL enrichment LG, called LG15, and QTLs of diameter, body wet weight and gonad wet weight shared the same region on LG15 (Table 4), which suggested that pleiotropism might generally exist on LG15. QTL enrichment has been proven extensively within plants^28–30^. Although many QTLs were mapped in other aquatic species^31–33^, no QTL enrichment for multiple traits has been reported. This paper reports QTL enrichment in aquatic species for the first time and supports our current high-density linkage map of sea urchin as a reliable reference for mapping important traits.

Distortions from expected Mendelian segregation are common among bivalves and other species^34,35^. Distorted markers were sometimes clustered in certain regions called segretation distortion region^36^. In this paper, 11 out of 13 extremely distorted SLAF markers spanned 12 cM continuously on LG16 (Supplementary Figure 3), indicating that a segretation distortion region might imply in it. Plough *et al*. reported that LG2 showed the most extreme distortion of ten LGs in wild Pacific oysters^13^. Here, we also found this phenomenon and the SLAF markers enriched in LG16 and LG19. Interestingly, no QTLs responsible for the eight economic traits were detected in these two LGs. In marine invertebrate species, a widely reported opinion explaining SD is that Mendelian segregation occurs during early developmental stages but accumulates to distorted during later development^37,38^. These results suggested that not certain reproductive traits but some traits accumulated mutation through mitosis during later development might associated with SD.

HRM is an effective modern method for genotyping and has expanded considerably in recent years^39–41^. Several SNPs located in QTL regions were experimentally validated with HRM in this paper, and an informative SNP potential for MAS breeding application was screened. We found that the NGS genotyping results were consistent with HRM genotyping and that SNP-29 was a functional marker for MAS breeding. These results show that HRM is an efficient approach for SNP genotyping.

Many QTLs were identified in aquatic species, but few could be used for molecular breeding applications. In this study, three SNPs were genotyped in an additional three sea urchin families and SNP-29, which explained 11.20% and 14.10% of the phenotypic variation for test diameter and body weight, respectively; these showed significant relationships with economic traits, indicating that multiple genes closely linked with SNP-29 were co-separated with economic traits without genetic background bias. SNP-29 can be genotyped by HRM, which is rapid and cost-effective for genotyping^42^. This means that SNP-29 is an effective functional marker and promising for MAS breeding in the future.

## Materials and Methods

### Materials and phenotyping

Sea urchins were hatched and raised at the Key Laboratory of Mariculture & Stock Enhancement in North China Sea, Ministry of Agriculture, Dalian Ocean University, China. An F_1_ genetic segregation population of sea urchin was generated by a cross between No.15 (male) and No.2–4 (female) in March 2014. Each offspring individual was bred in a special cage, and abundant fresh kelp was added to the cage regularly. All experimental cages of sea urchins were fed the same diet to avoid variation caused by environmental factors. Aerobic equipment was checked every day to ensure normal oxygen supply. Seawater was changed according to its quality and sea urchin life state. Water temperature, pH and salinity were recorded before and after sea water change. During the experiment, water temperature ranged from 8 °C to 24 °C, pH ranged from 7.9 to 8.0, and salinity ranged from 30 to 31. Eventually, a total of 150 randomly selected individuals were used for phenotyping and genotyping. Eight quantitative traits were measured in August 2015. Test height, test diameter, body weight and Aristotle’s lantern weight were measured by conventional methods. The entire gonad was removed carefully without damage and weighed, blotted dry and weighed again. Dry gonad rate was calculated following this formula: Dry gonad weight/fresh gonad weight * 100%. Light orange-yellow color difference (Δ*E*_*1*_) and light yellow color difference (Δ*E*_*2*_) were measured using our previous research method^43^. Three replicate measurements of light orange-yellow color difference (Δ*E*_*1*_) and light yellow color difference (Δ*E*_*2*_) per sample were averaged to give a single measurement.

All phenotypic statistical analyses were conducted with ANOVA analysis using SPSS 16.0 software. The Kolmogorov-Smirnov Test was used for the normal test.

### DNA extraction

After weight measurements, the esophagi of 152 samples, including two parents and 150 F_1_ individuals, were collected separately, frozen in liquid nitrogen immediately, and transferred to a -80 °C freezer. Total genomic DNA was extracted from each esophagus sample following the cetyltrimethyl ammonium bromide (CTAB) method^44^. The concentration and quality of extracted DNA were examined by electrophoresis in a 1% agarose gel with a standard lambda DNA and an ND-2000 spectrophotometer (NanoDrop, Wilmington, DE, USA).

### SLAF library preparation and sequencing

*Rsa*I and *Hae*III (New England Biolabs, NEB, USA) were used to digest the genomic DNA of the F_1_ population and the two parents. A modified SLAF-seq strategy was utilized in our experiment. Tian Liu *et al*.'s method was followed, but fragments ranging from 414 bp to 464 bp base pairs (with indexes and adaptors) were excised and purified. Gel-purified products were diluted properly and loaded on an Illumina HiSeq. 2500 system for pair-end sequencing (100 bp per end) (Illumina, Inc., San Diego, CA, USA) according to the manufacturer’s recommendations at Biomarker Technologies Corporation in Beijing (http://Biomarker.com.cn/). Q30 (a quality score of 30; indicating a 0.1% chance of an error, and thus 99.90% confidence) and GC content of the raw data were adopted as standards for data quality control. Raw sequence reads were deposited in the NCBI-short read archive (SRA) database (accession: SRR4426280).

### SLAF-seq data analysis and genotyping

SLAF-seq data was performed using procedures described by Sun *et al*.^45^. Reads were quality-filtered by requiring at most Q20 < 20e and the remaining raw reads were sorted to each progeny according to duplex barcode sequences. The barcodes and terminal 5-bp positions were trimmed from each high-quality read, leading to clean reads. Reads with similarity = 90% and meanscore = 45 by BLAT were grouped to one SLAF locus^46^. Single nucleotide polymorphism (SNP) loci of each SLAF locus were detected between parents, and SLAF-tags with more than three SNPs were filtered out first. Alleles were defined in each SLAF using minor allele frequency (MAF). In the mapping populations of diploid sea urchin, one locus can contain no more than four SLAF tags, and thus groups with more than four tags were considered repetitive SLAFs and excluded. Polymorphic SLAFs, which refer to SLAFs with 2–4 different tags, were considered potential markers for the next genetic map construction. These polymorphic SLAF markers were coded into the eight segregation patterns ab × cd, ef × eg, hk × hk, lm × ll, nn × np, aa × bb, ab × cc and cc × ab.

### Genetic map construction and QTL analysis

To ensure a high-quality genetic map, we conducted four quality control steps for the polymorphic SLAF marker except aaxbb type: 1) the average depths of each parent were below 10-fold; 2) a SLAF that interspersed more than five SNP; 3) segregating markers could be genotyped in at most 70% of individuals; 4) markers with significant SD (*P* < 0.01). The other SLAF markers were used to perform linkage map construction. HighMap software^12^ was used to construct the genetic map of sea urchin after linkage grouping according to single-linkage clustering algorithm at the logarithm of odds (LOD) threshold ≥5.0 and a maximum recombination rate of 0.4. We employed a Kosambi mapping function to convert recombination percentages to genetic distance (cM). Haplotype maps and heat maps were also used to evaluate the quality of the constructed linkage map^12^.

QTL analysis was conducted employing MapQTL V5.0 software using an interval mapping (IM) model. The significance thresholds were determined using 1,000 permutations. Calculation of the percentage of phenotype variance explained by each QTL (Expl. %) was achieved based on the population variance within the segregation population.

### Primer design and HRM analysis

Primers based on SNP were designed by Primer 5.0 according to SLAF marker sequences resulting from QTL region falling into genes with annotation information from the *Strongylocentrotus purpuratus* genome. Criteria for PCR primer design includes a predicted annealing temperature (Tm) of 50–60 °C, limited self-complementarity and poly-X, and PCR amplicon lengths of 40–120 bp. Gradient PCR running was conducted to obtain optimal Tm values. Every primer pair included only one SNP locus and no InDel locus was tolerated.

PCR performed in a 10-μL final volume contained 20 ng DNA (10 ng/ul), 2.5 × Light Scanner Master Mix 4 µl, 1 µl of each primer (10 µmol/L), and 1 µl of each internal standard primer (high temperature and low temperature). Cycling conditions were 95 °C for 5 min, 35 cycles at 94 °C for 30 s, annealing for 30 s, 72 °C for 30 s, 5 min at 72 °C for elongation and 4 °C for conservation. PCR products were examined by 4% agarose gel electrophoresis. The primers for genotyping analysis were gathered in accordance with a preliminary HRM assay to ensure sure the optimal annealing temperature and amplicons with a unimodal curve between high and low temperature internal standard curves, which were conserved for genotyping. The internal standard primer information is displayed in Supplementary Table 6. Using a Light Scanner96 (Idaho Technology Inc., Salt Lake City, Utah, USA), 48 randomly chosen individuals from 150 F_1_ offspring were subjected to HRM analysis.

To verify the SNP effect screened above and assess its utilization potential for MAS breeding, three additional sea urchin families with significant difference to corresponding growth-related traits were also genotyped by HRM. Thirty individuals per family were randomly selected. DNA extraction, phenotyping, HRM and other related operations were as mentioned above.

## Electronic supplementary material

Supplementary Information
