# Supplementary material for: SLAF-based high-density genetic map construction and QTL mapping for major economic traits in sea urchin Strongylocentrotus intermedius
Source: Sci Rep. 2018 Jan 16;8:820. doi: 10.1038/s41598-017-18768-y (PMC5770408; doi:10.1038/s41598-017-18768-y)
Supplement: Supplementary file 1 — Supplementary Information [file 41598_2017_18768_MOESM1_ESM.pdf]

SLAF-based high-density genetic map construction and QTL mapping for major economic traits in sea urchin *Strongylocentrotus intermedius*

Yaqing Chang1*†, Jun Ding1†, Yuhui Xu2†, Dan Li1, Weijie Zhang1, Lei Li1, Jian Song1

*Corresponding author: Yaqing Chang

Dalian Ocean University, Dalian China.

Fax: +86-0411-84762691.

E-mail addresses: yaqingchang@hotmail.com

†Equal contributors

1Dalian Ocean University, Key Laboratory of Mariculture & Stock Enhancement in North China's Sea, Ministry of Agriculture, Dalian Liaoning 116023,Chian.

2Biomarker technology Corporation, Beijing 101300, China.

Additional Information

Sample ID	SLAF number	Total depth	Average depth
Male	249,076	8,125,594	32.62
Female	279,416	5,737,786	20.53
Offspring	190,361	1,675,018	8.80

Supplementary Table 1. Statistics for SLAF tags.

Linkage Group	Marker number	Linkage Group	Marker number
1	4	12	5
2	8	13	2
3	0	14	2
4	6	15	1
5	3	16	13
6	1	17	0
7	4	18	1
8	9	19	12
9	0	20	8
10	3	21	3
11	0	-	-

Supplementary Table 2. Statistics for SD markers.

Linkage Group	Double crossover rate (%)
1	0.00
2	0.01
3	0.05
4	0.11
5	0.04
6	0.01
7	0.16
8	0.00
9	0.02
10	0.11
11	0.16
12	0.83
13	0.02
14	0.01
15	0.21
16	0.04
17	0.08
18	0.16
19	0.02
20	0.07
21	0.07

Supplementary Table 3. The double crossover rate across the genome.

SNP ID	SLAF ID	Related traits	LOD	Contribution R^2(%)	Forward primer (5'-3')	Reverse primer (5'-3')	SNP type	Product length(bp)
SNP-22	17945	Aristotle's lantern weight	3.14	11	GTGTAAGACAGAGTCGGAAT	CGTCAATGAGGGTCTACC	T/C	67
SNP-29	19255	Test diameter	3.49	11.2	CAGGTATCCTAAAGCAAT	GTTTGTTGGTAAGCACTG	T/C	44
SNP-29	19255	Body weight	4.47	14.1	CAGGTATCCTAAAGCAAT	GTTTGTTGGTAAGCACTG	T/C	44
SNP-37	85700	Test diameter	3.19	10.3	CTCAAGTCCCACCACAAC	GAATACAGATGAGTGGCTTG	A/C	51

Supplementary Table 4. QTL mapping information of 3 SNP loci and primer information.

	SNP-22		SNP-29		SNP-37	
Traits	Homozygous genotype	Heterozygous genotype	Homozygous genotype	Heterozygous genotype	Homozygous genotype	Heterozygous genotype
Sample No.	70	17	59	31	73	17
Test height (mm)	19.45±4.66^{a}	21.46±2.17^{b}	18.51±4.47^{A}	22.45±2.38^{B}	19.84±4.56	19.99±3.05
Test diameter (mm)	36.89±11.00	40.29±4.96	34.57±10.34^{A}	43.69±6.17^{B}	37.75±10.72	37.56±6.88
Body weight (g)	22.40±15.78	25.26±6.06	18.83±14.14^{A}	31.26±10.82^{B}	23.47±15.46	21.57±7.86

Supplementary Table 5. Correlation test between genotypes and phenotypic traits of 3 SNPs. Capital letter and lower case letter at the top-right corner means significant difference was detected between the two genotypes at $P<0.01$ and $P<0.05$, respectively.

Primer ID	Sequence (5'-3')	Tm (°C)	Product length (bp)
High temperature	F:GCGGTCAGTCGGCCTAGCGGTAGCCAGCTGCGGCACTGCGTGACGCTCAG	90.08	50
	R:CTGAGCGTCACGCAGTGCCGCAGCTGGCTACCGCTAGGCCGACTGACCGC		
Low temperature	F:ATCGTGATTTCTATAGTTATCTAAGTAGTTGGCATTAATAATTTCATTTT	72.7	50
	R:AAAATGAAATTATTAATGCCAACTACTTAGATAACTATAGAAATCACGAT		

Supplementary Table 6. Internal standard complementary double chain and annealing system.

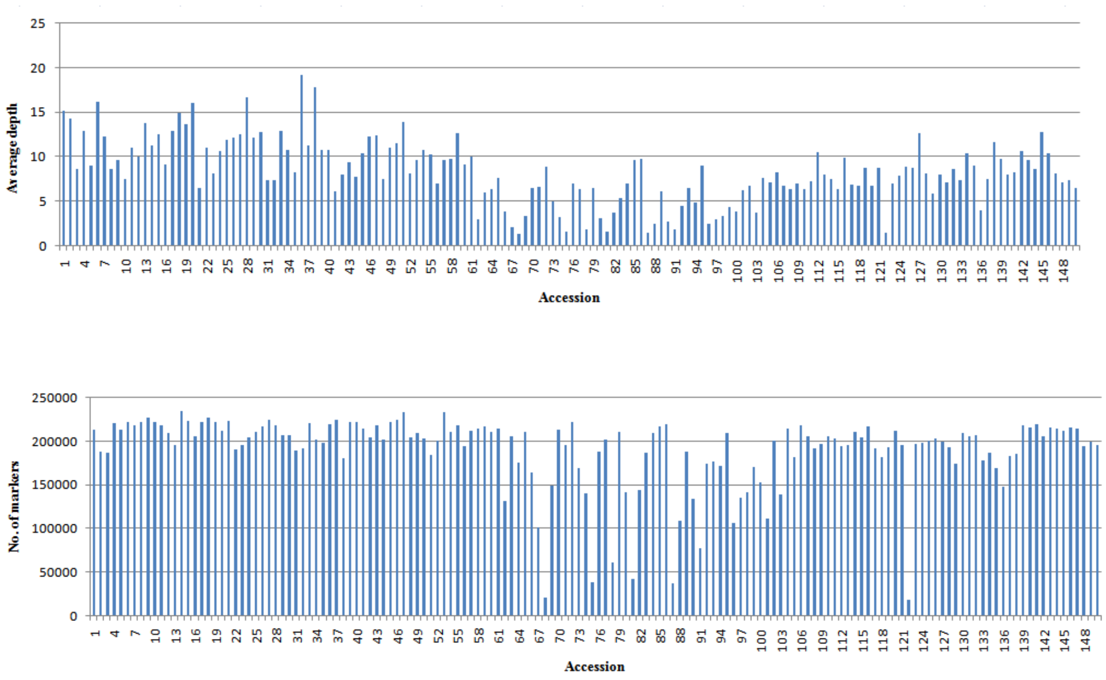

Supplementary Figure 1. Average depth and No. of SLAF markers of all accessions.

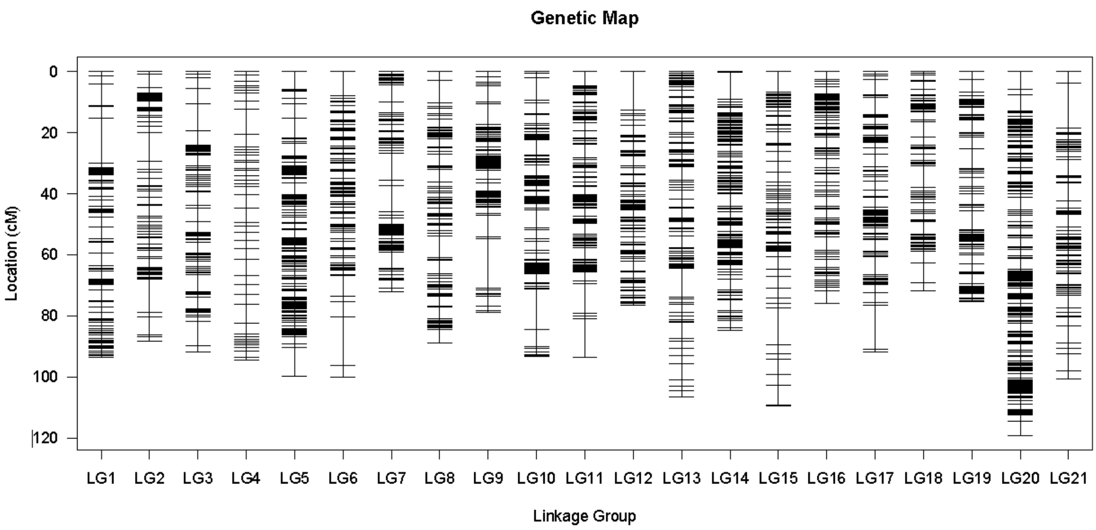

Supplementary Figure 2. View of a high-density genetic map of sea urchin (*Strongylocentrotus intermedius*).

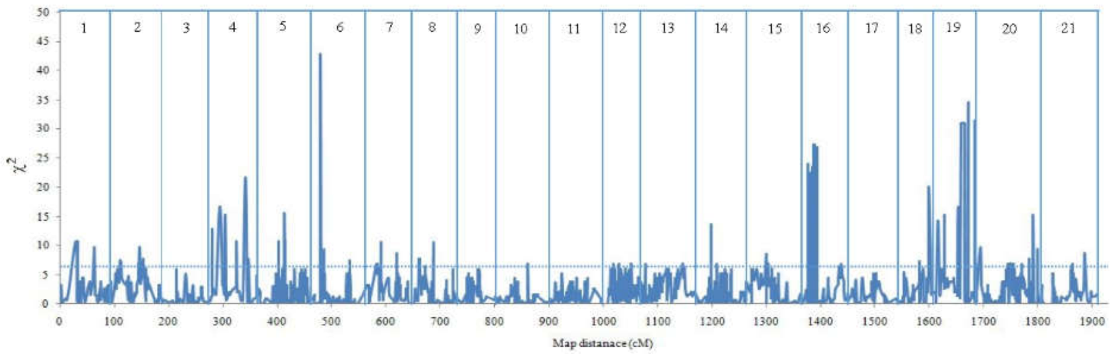

Supplementary Figure 3. Chi-square analysis of SLAF markers spanning on the genetic map.

The dotted light blue line indicates the threshold χ^2 value for extremely significance (*P*=0.01).

Number 1-21 between adjacent long vertical lines indicates the corresponding linkage group.

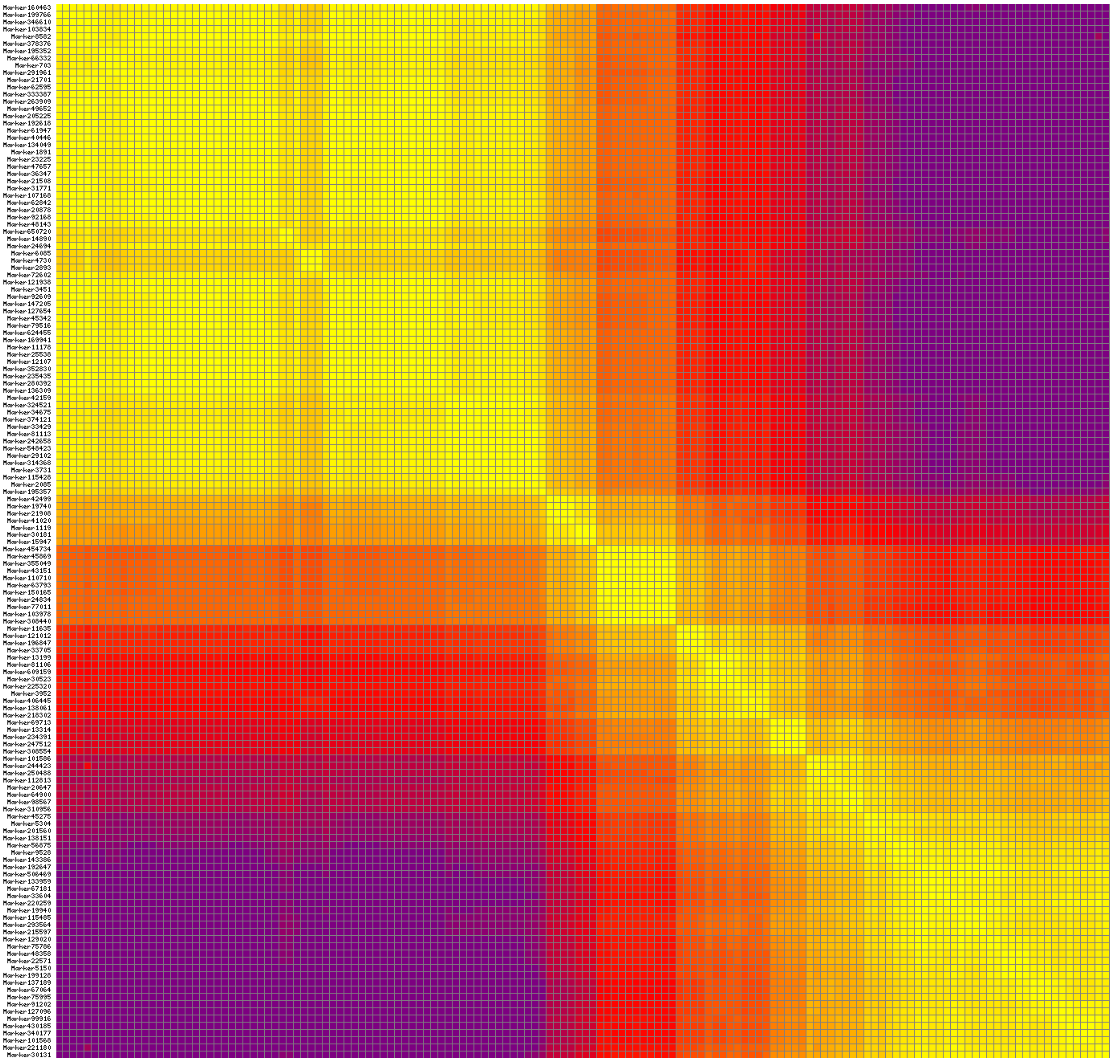

Heat map of LG1

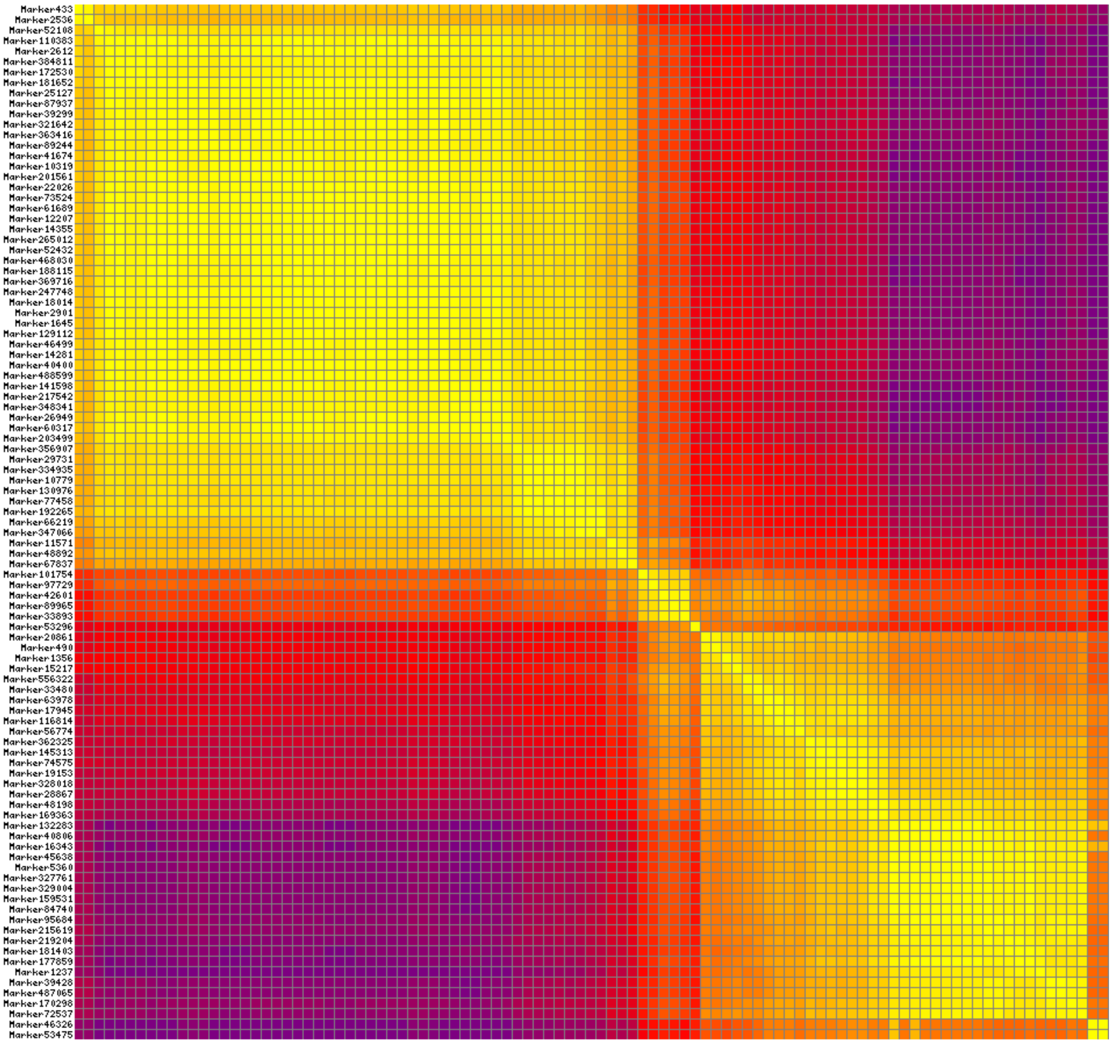

Heat map of LG2

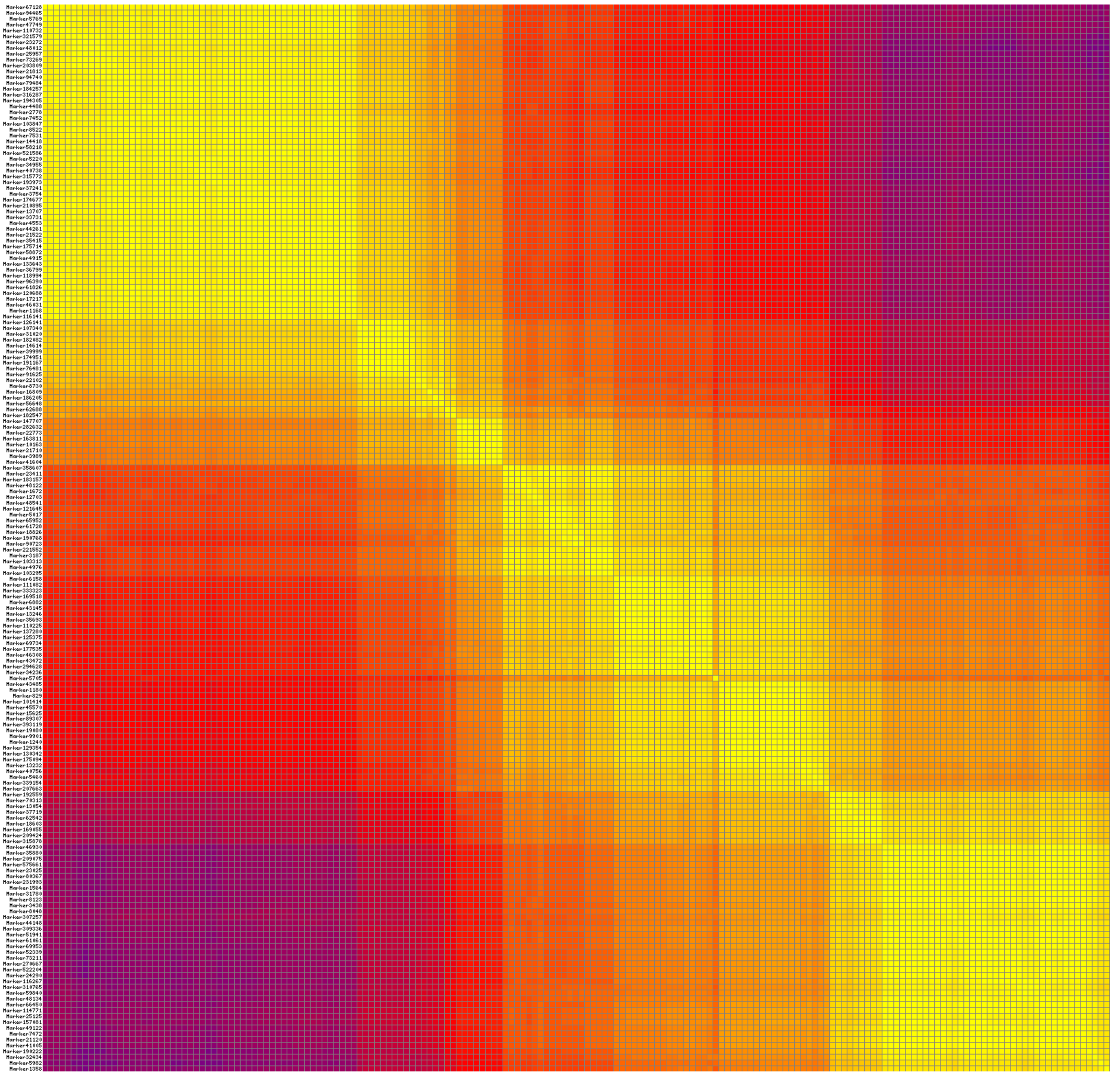

Heat map of LG3

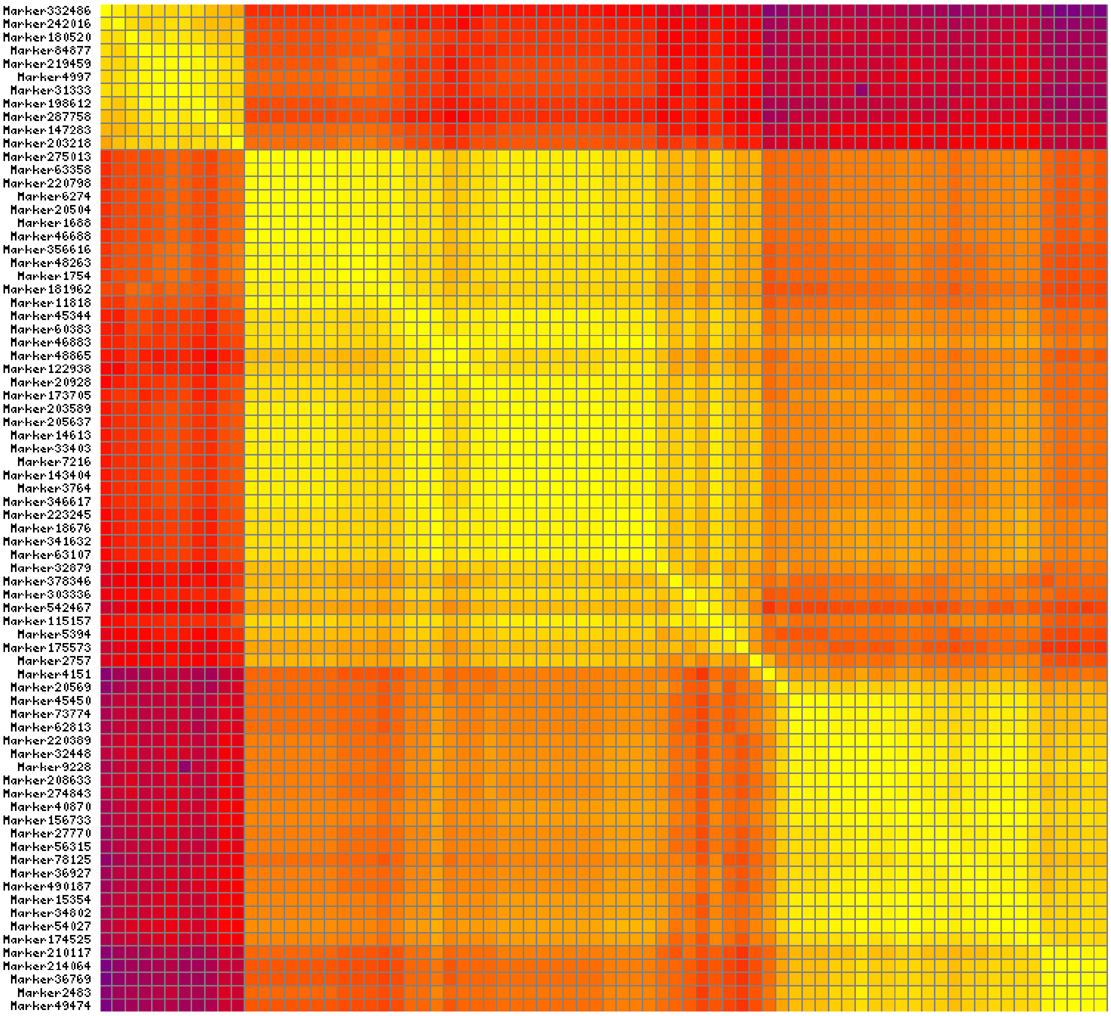

Heat map of LG4

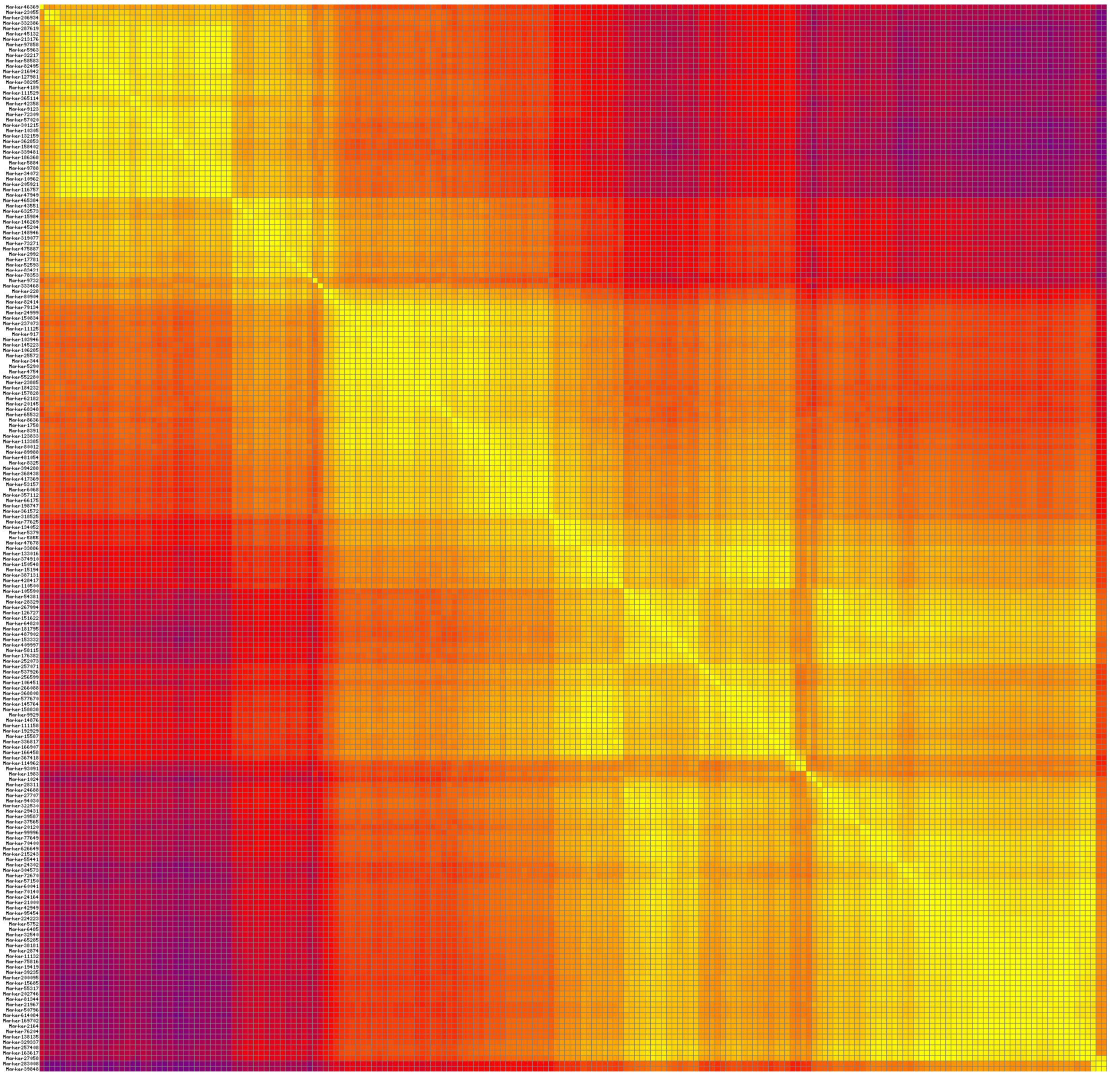

Heat map of LG5

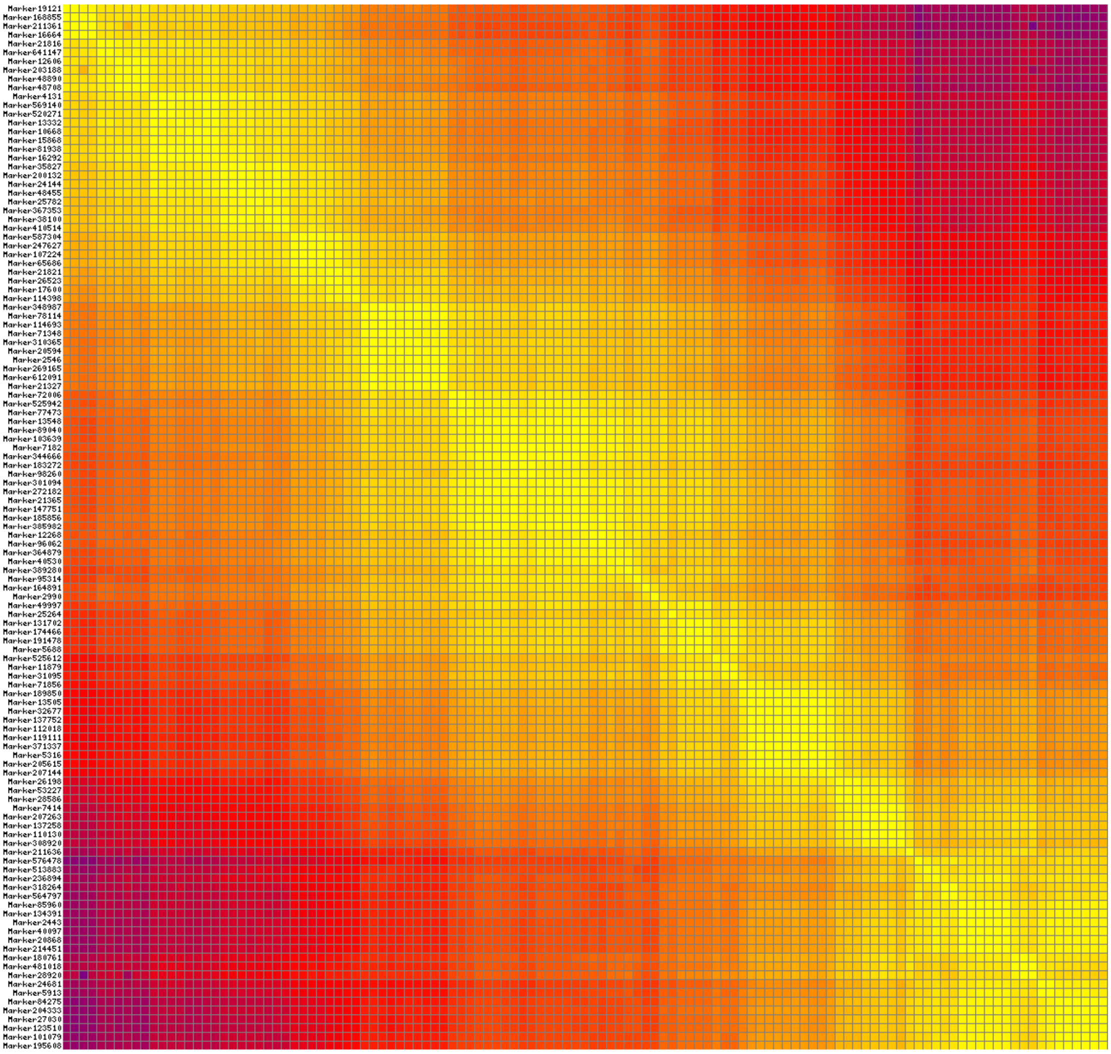

Heat map of LG6

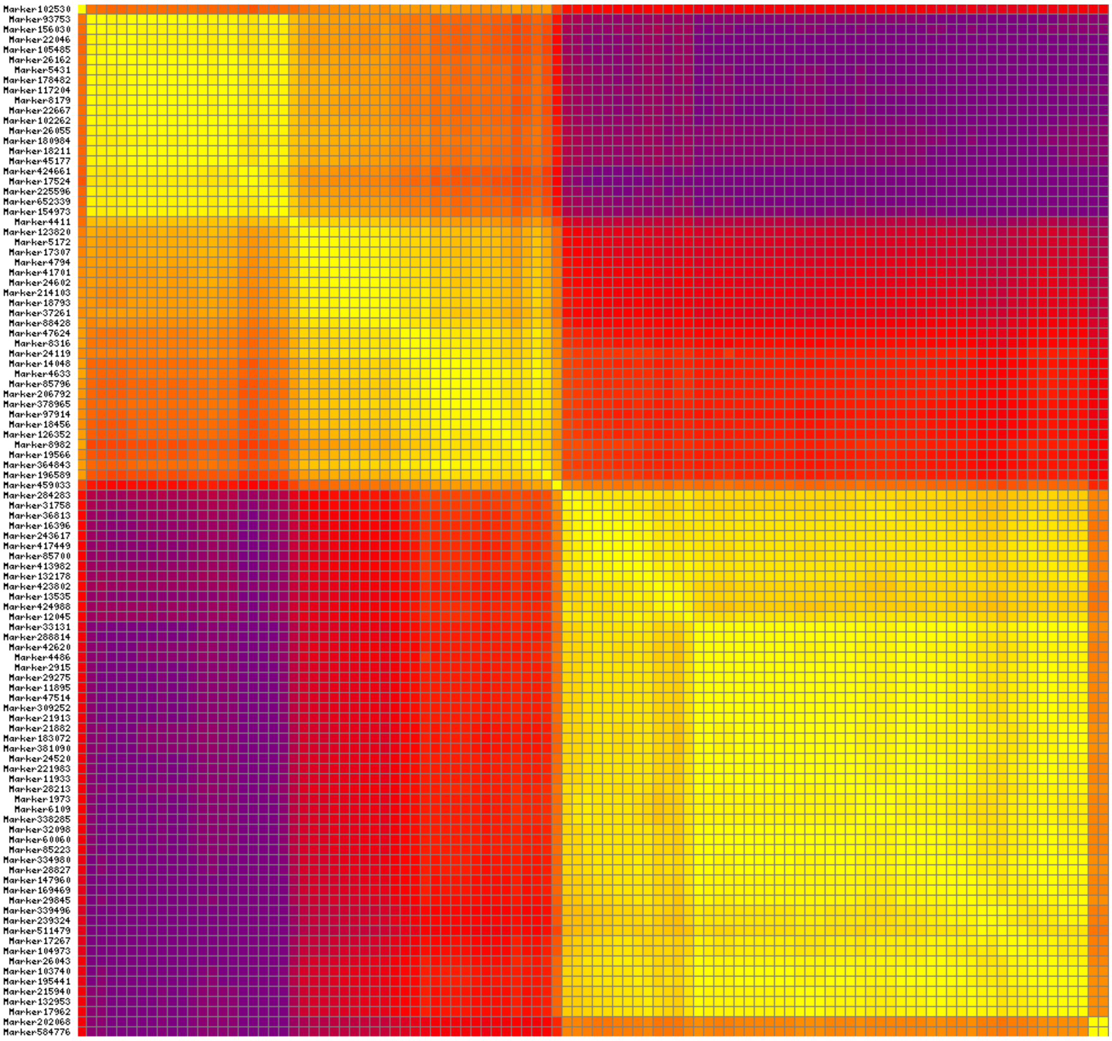

Heat map of LG7

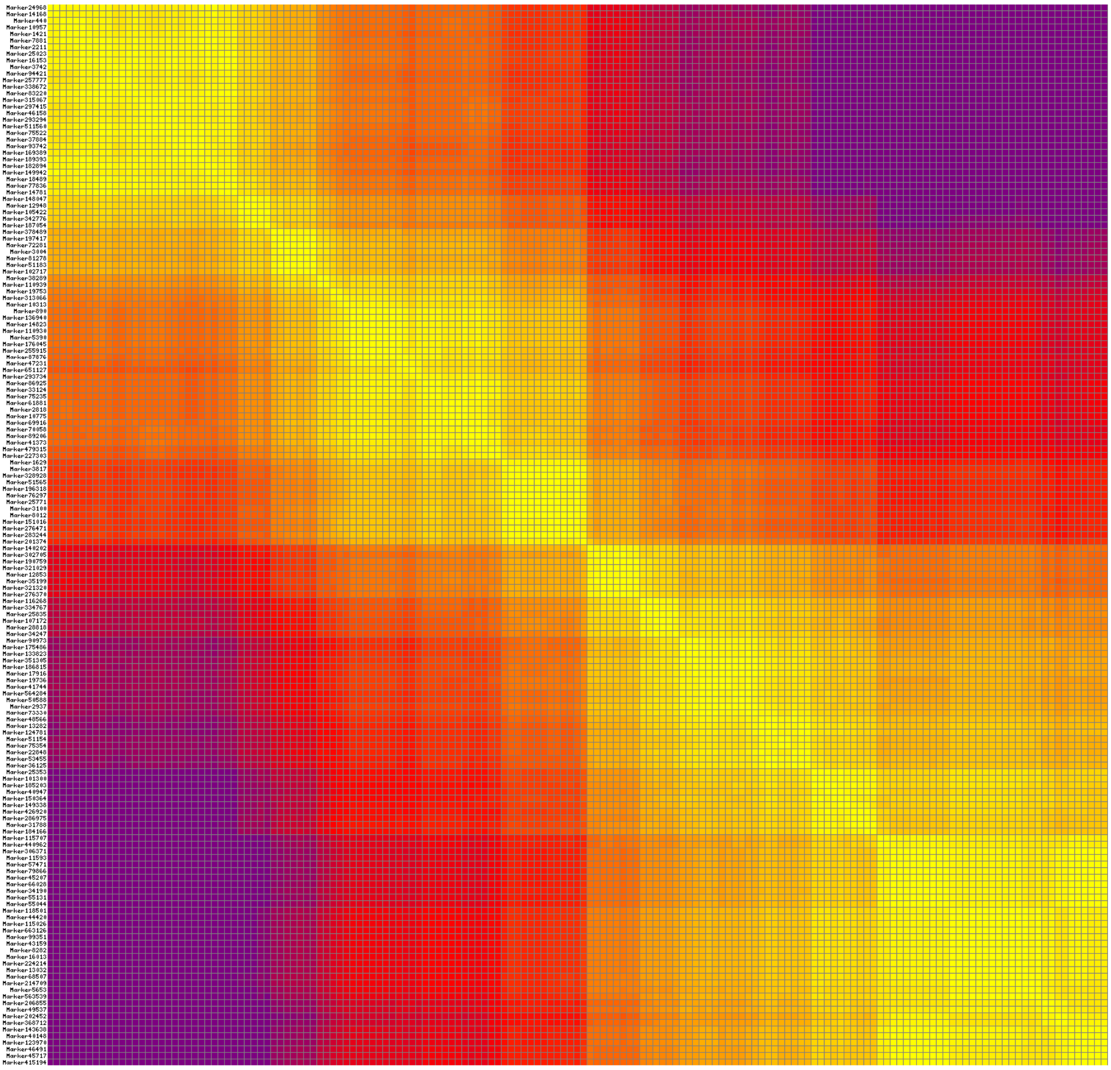

Heat map of LG8

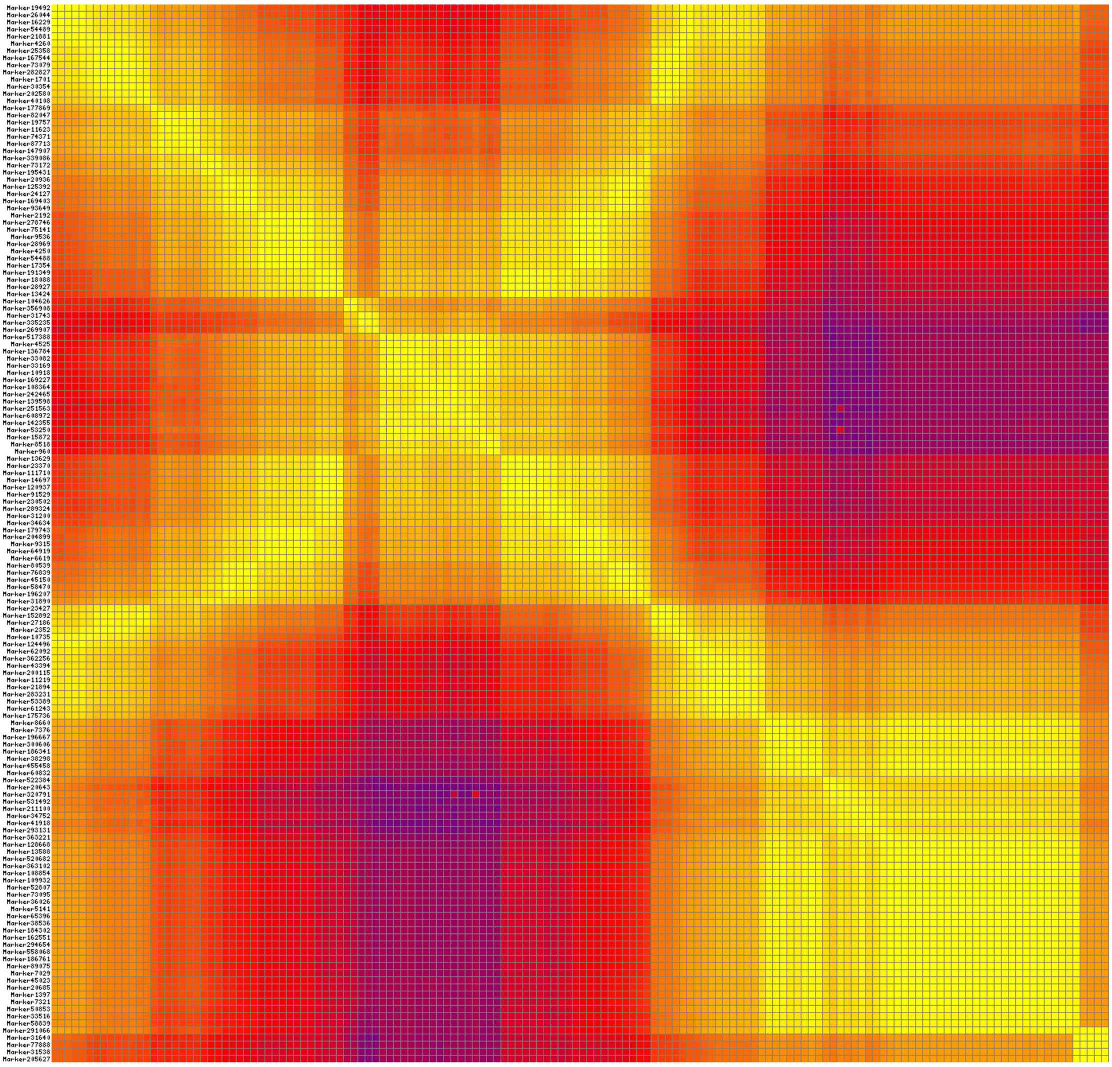

Heat map of LG9

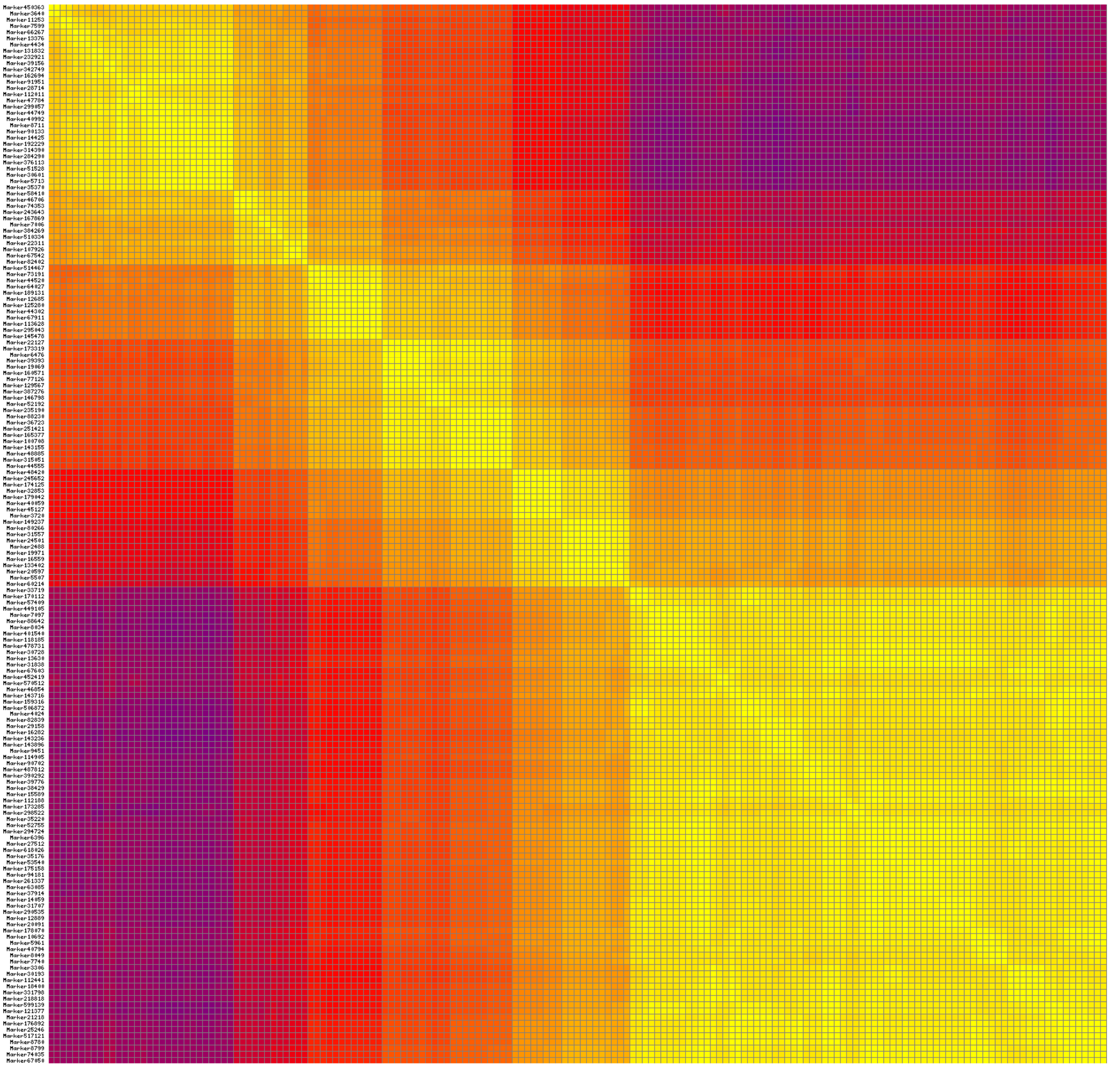

Heat map of LG10

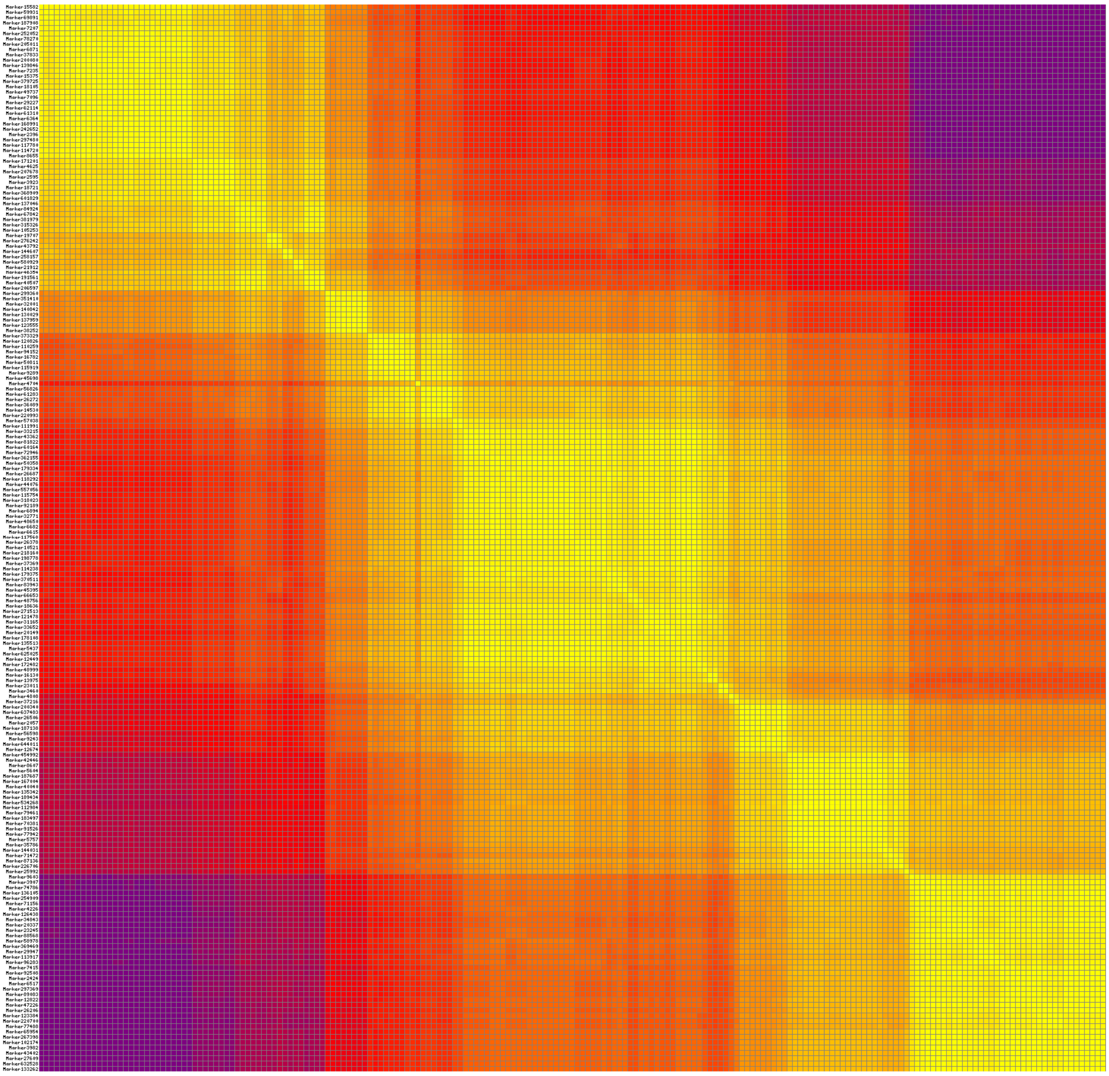

Heat map of LG11

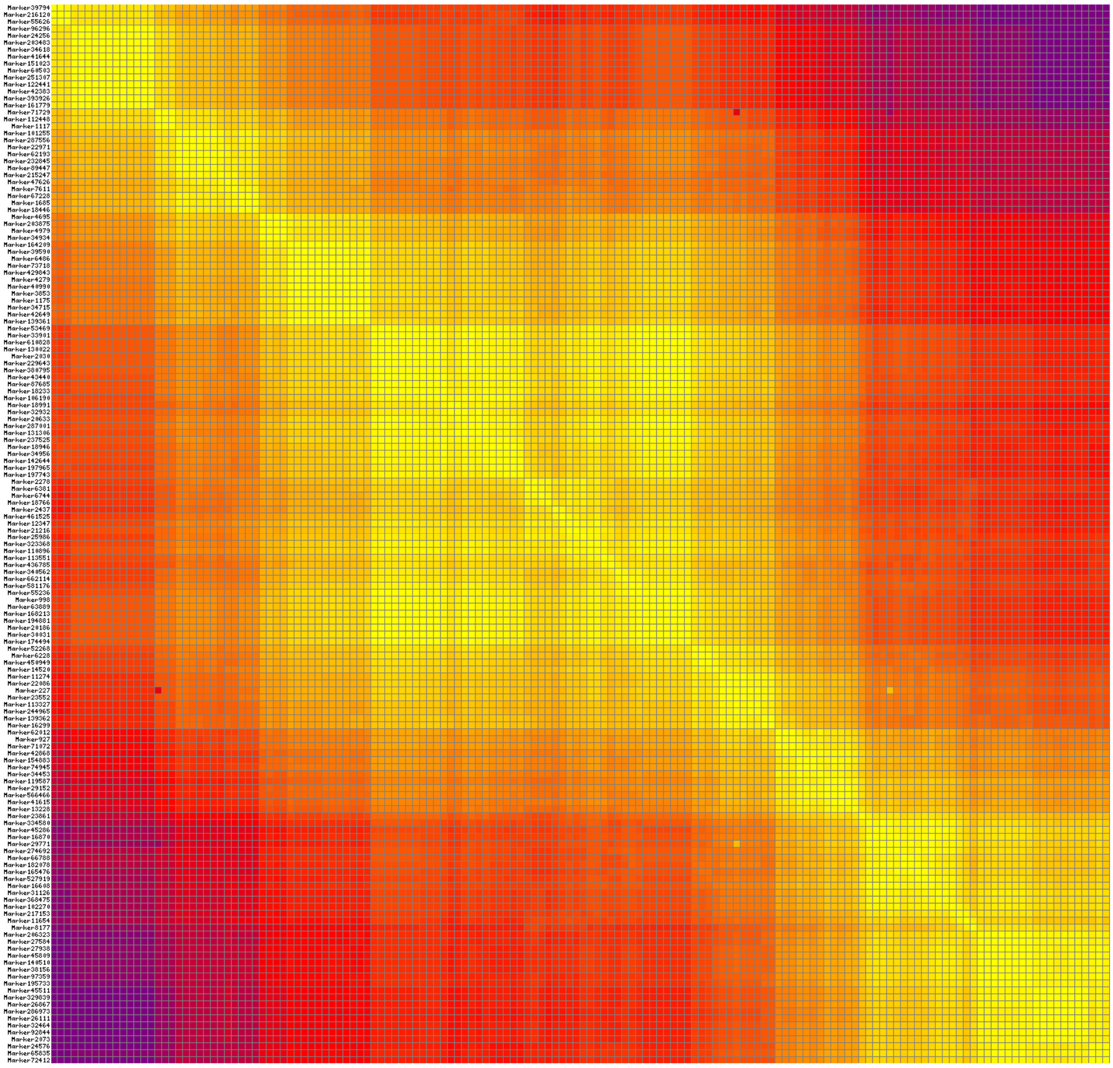

Heat map of LG12

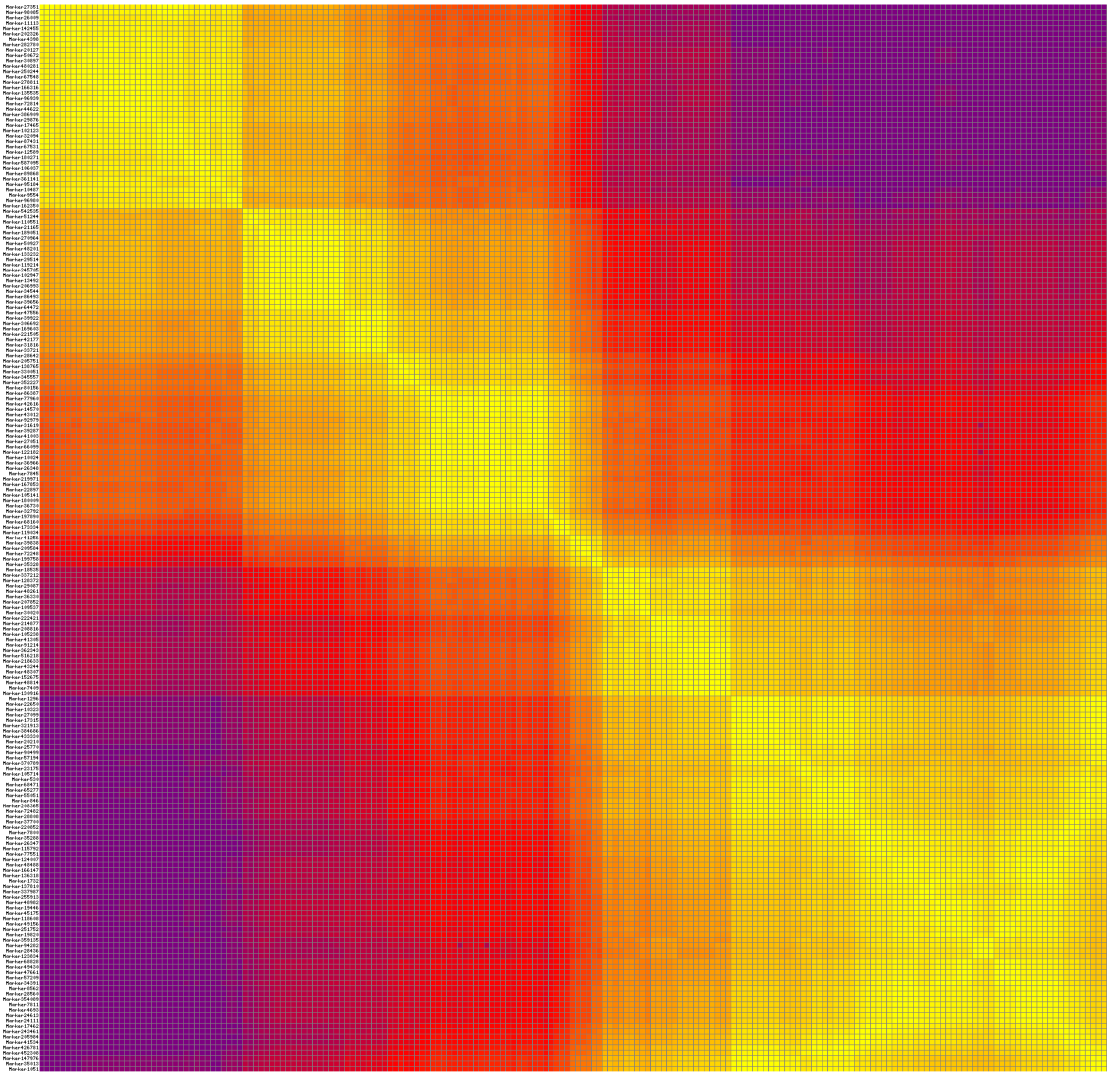

Heat map of LG13

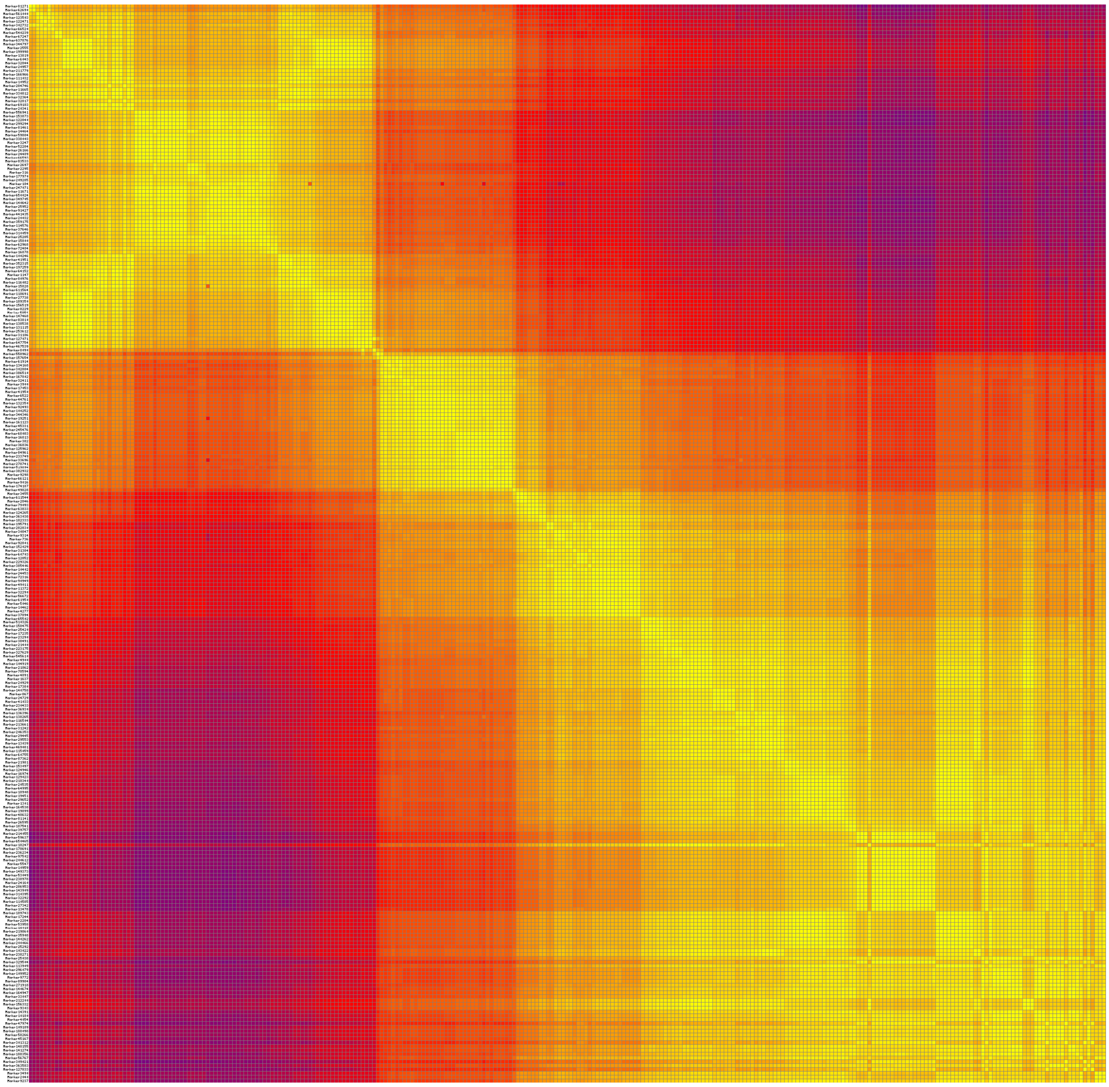

Heat map of LG14

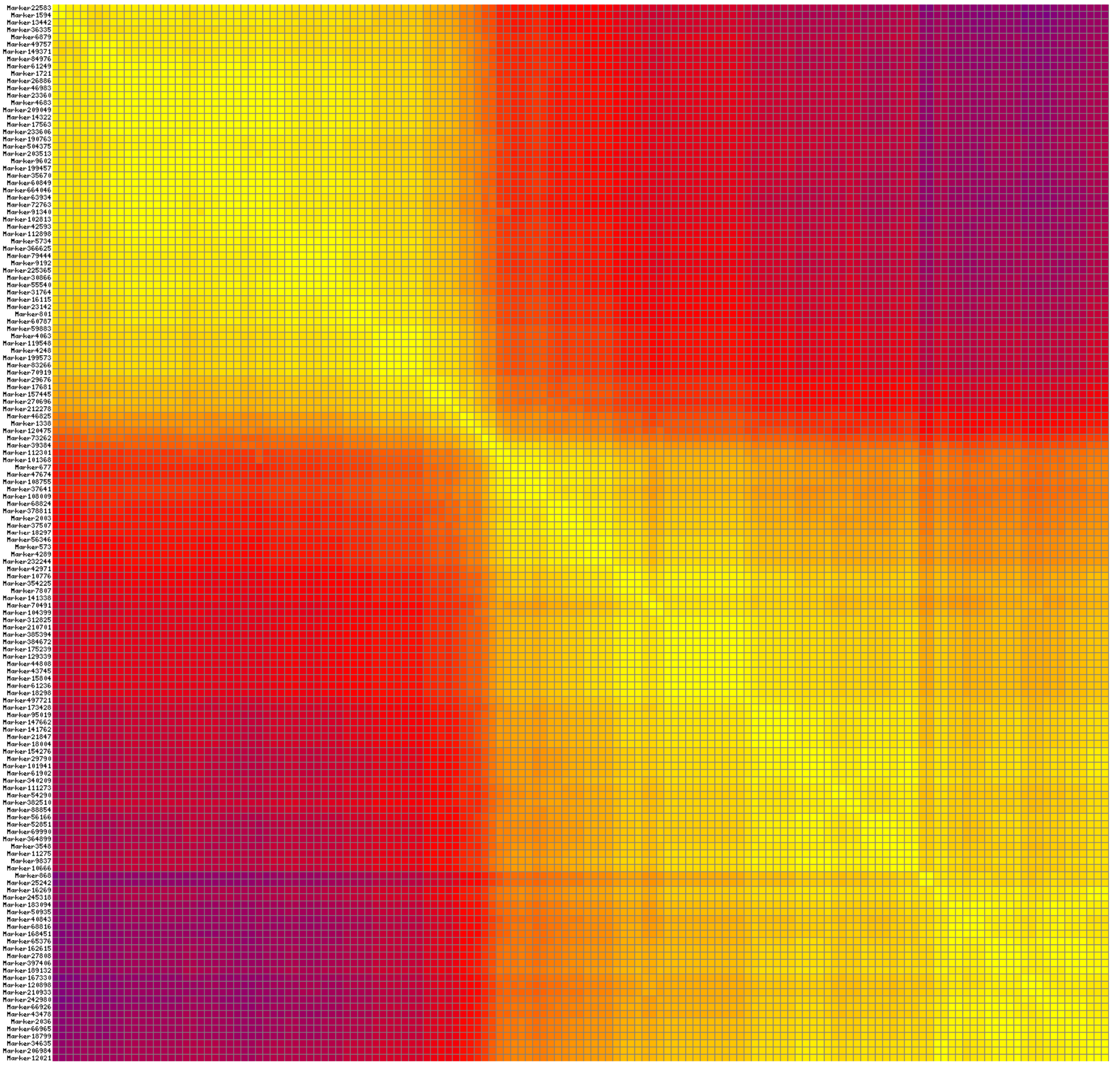

Heat map of LG15

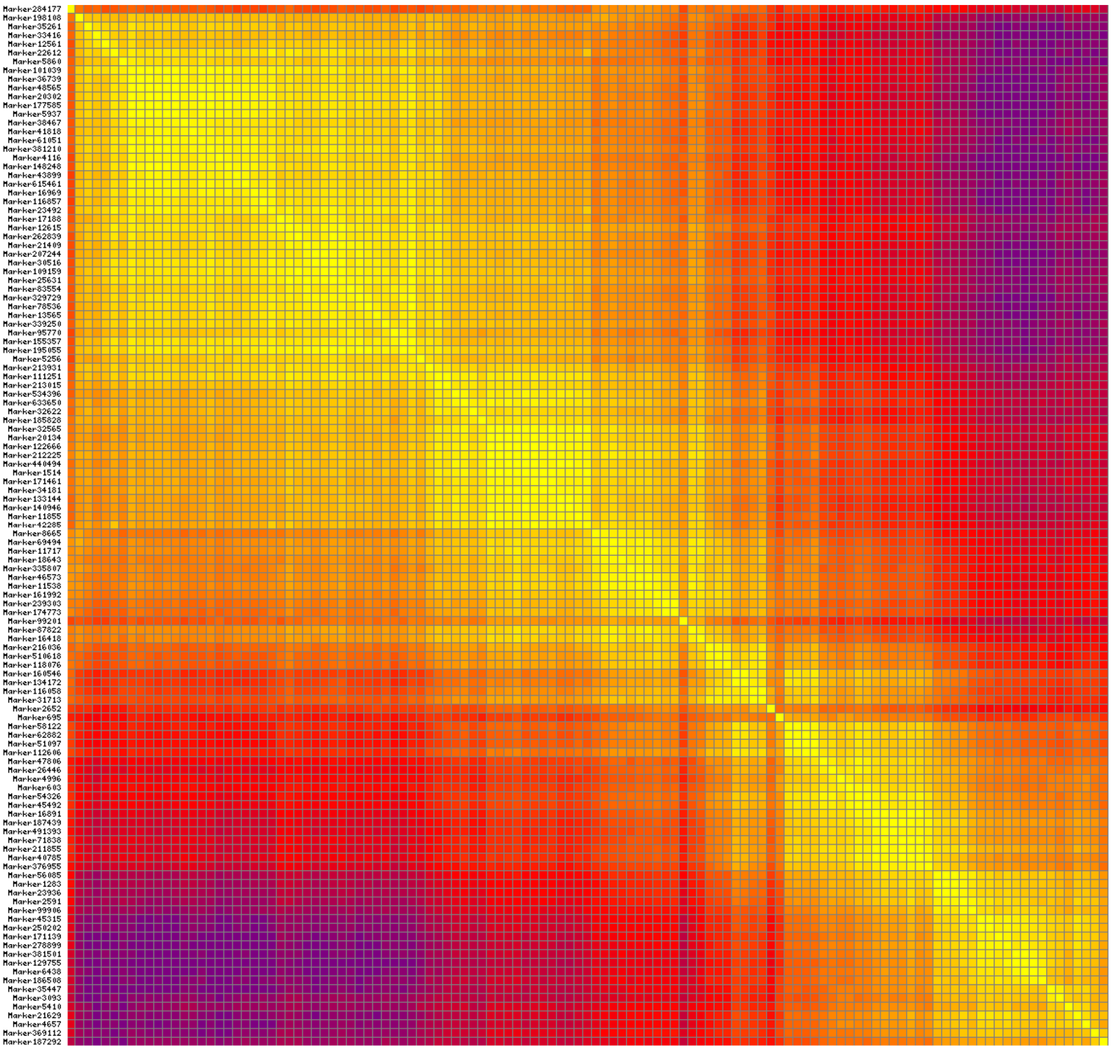

Heat map of LG16

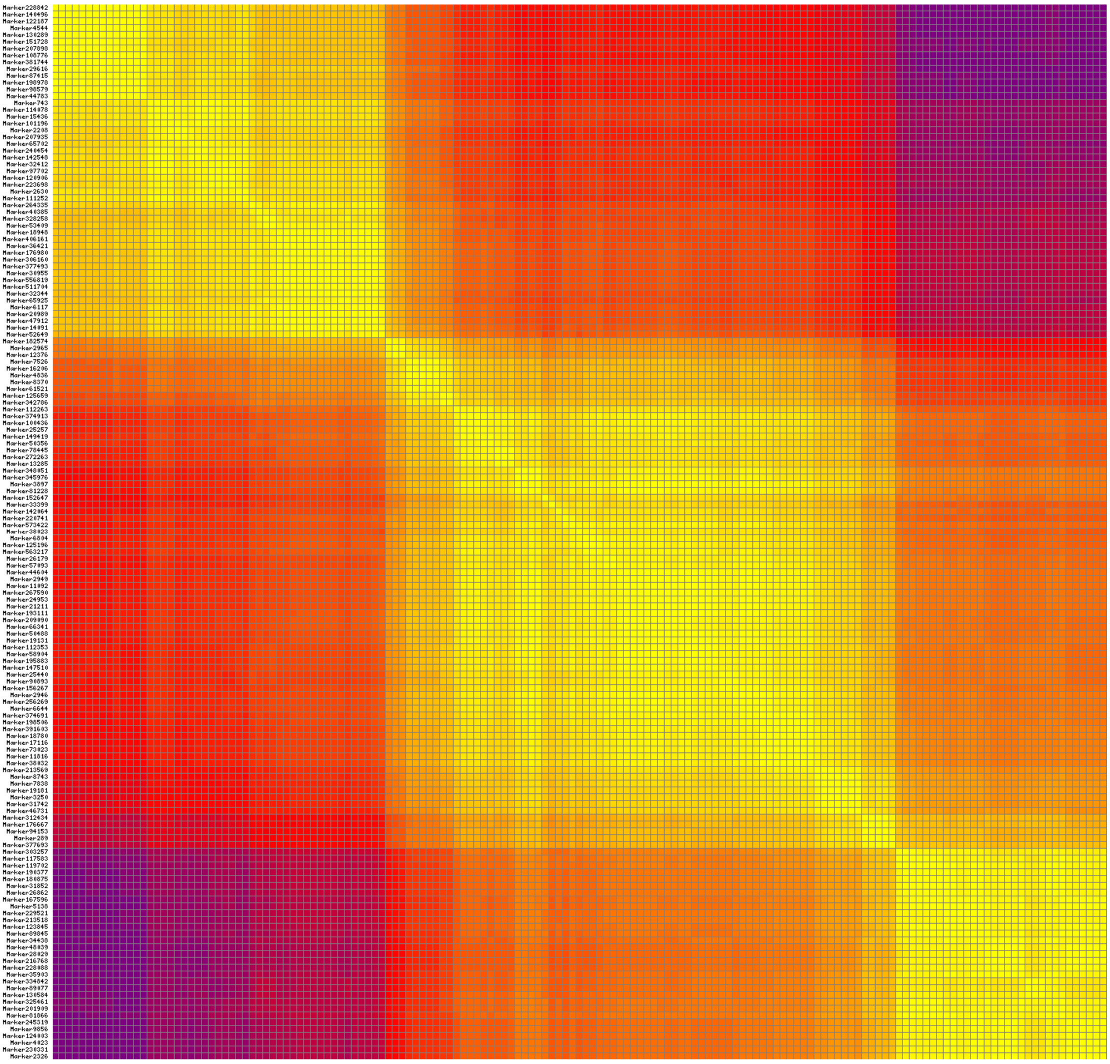

Heat map of LG17

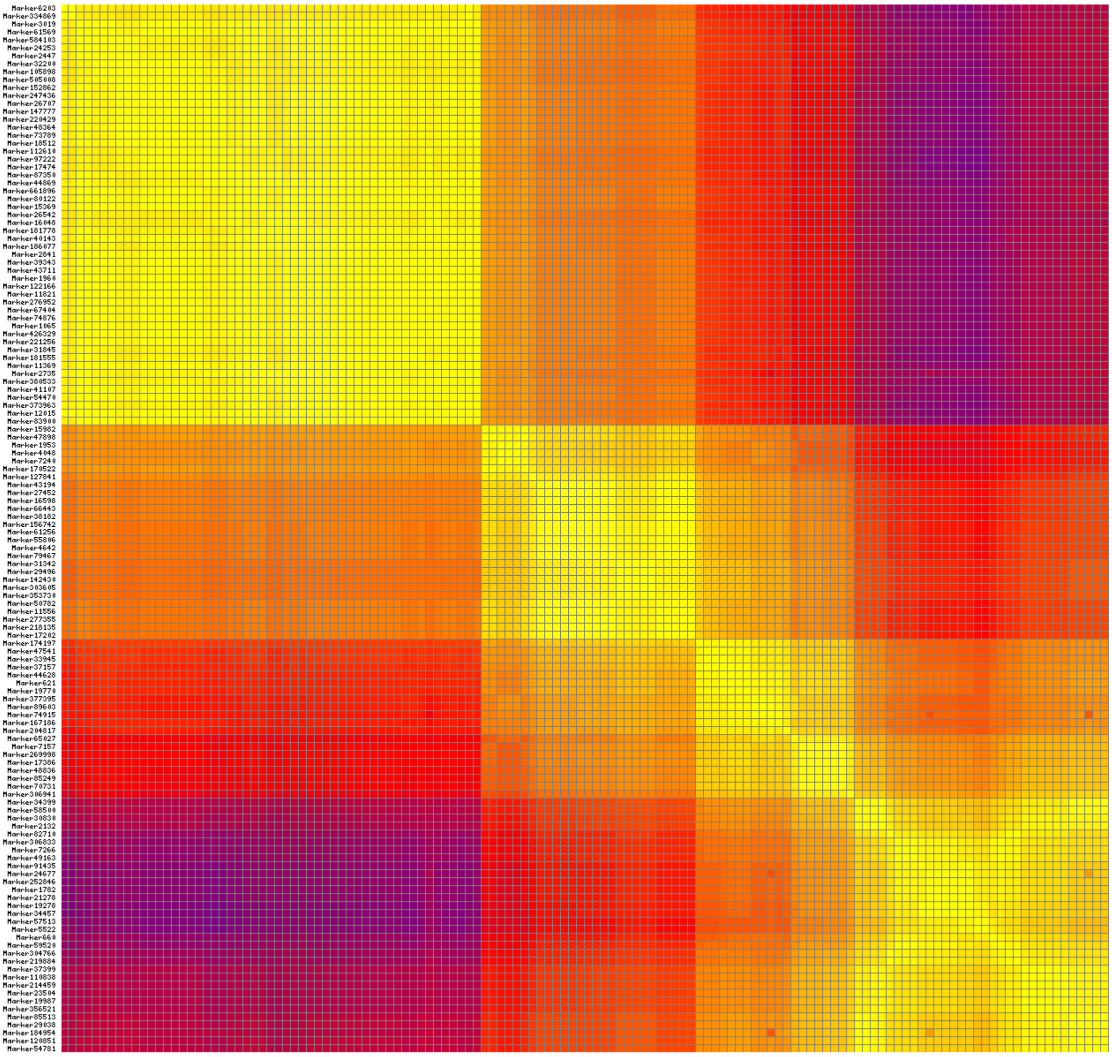

Heat map of LG18

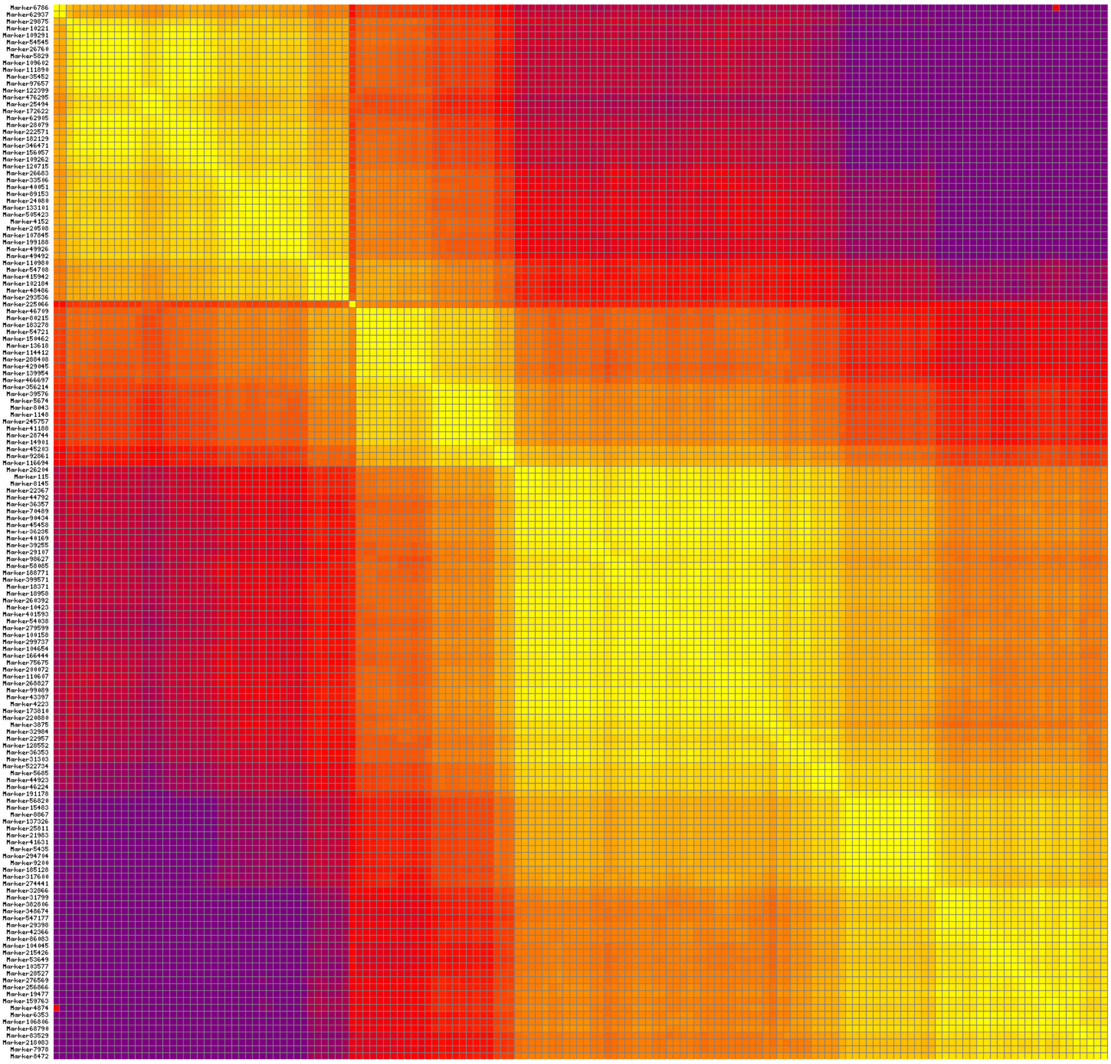

Heat map of LG19

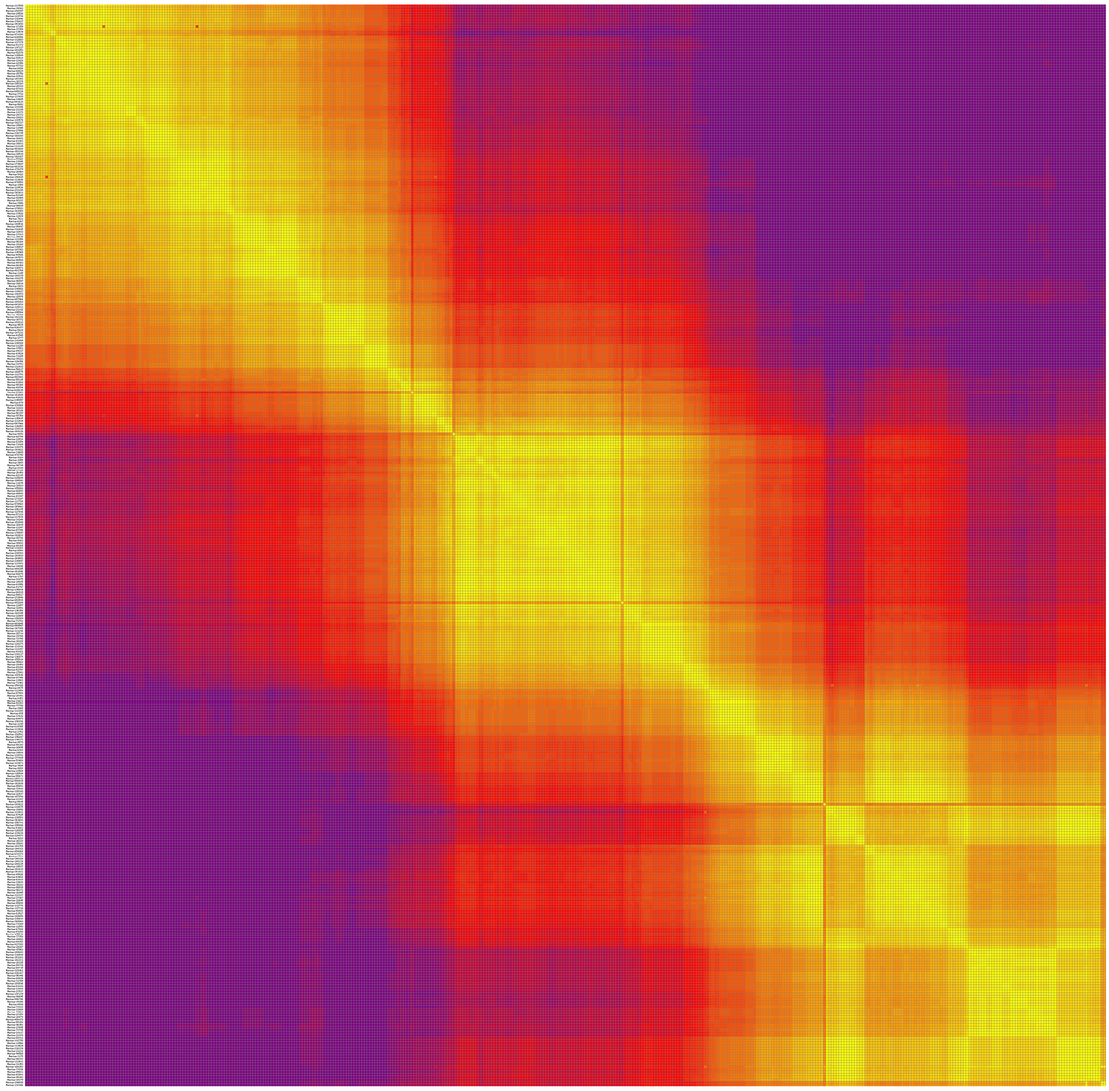

Heat map of LG20

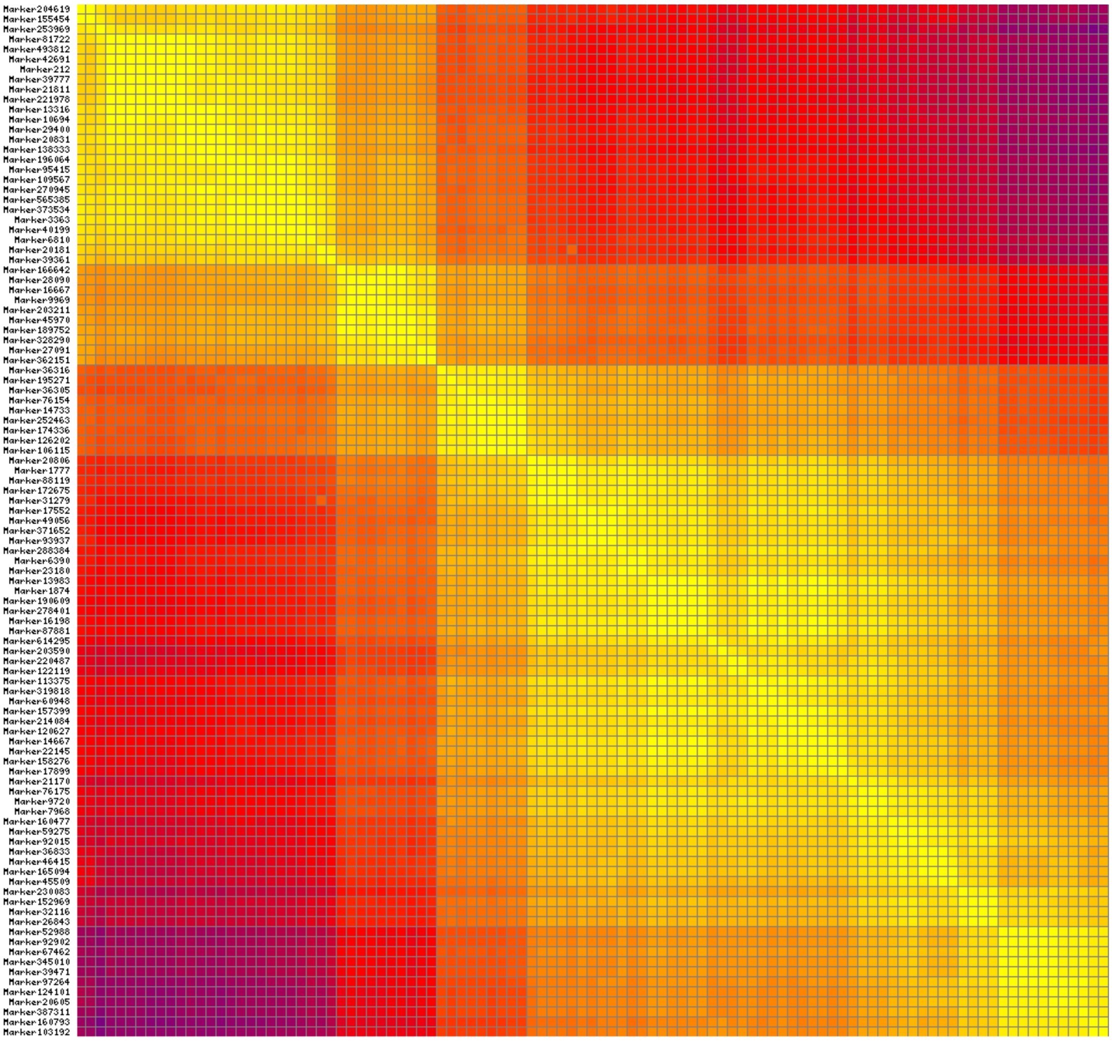

Heat map of LG21

Supplementary Figure 4. Heat maps of all LGs. Each cell represents the recombination rate of two markers. Yellow indicates a lower recombination rate and purple a higher one.

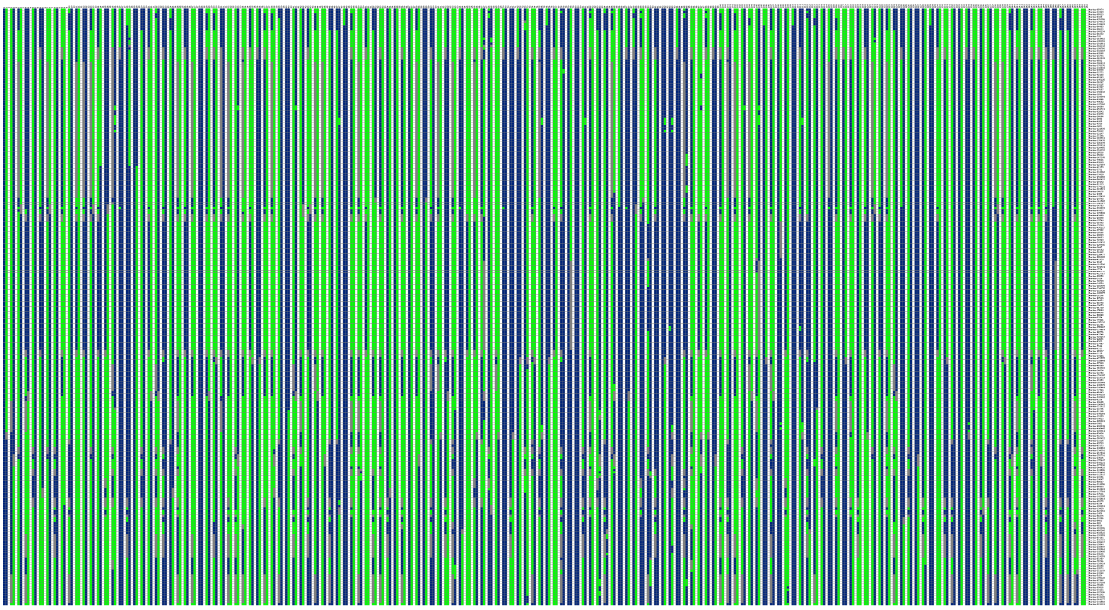

Haplotype map of LG1

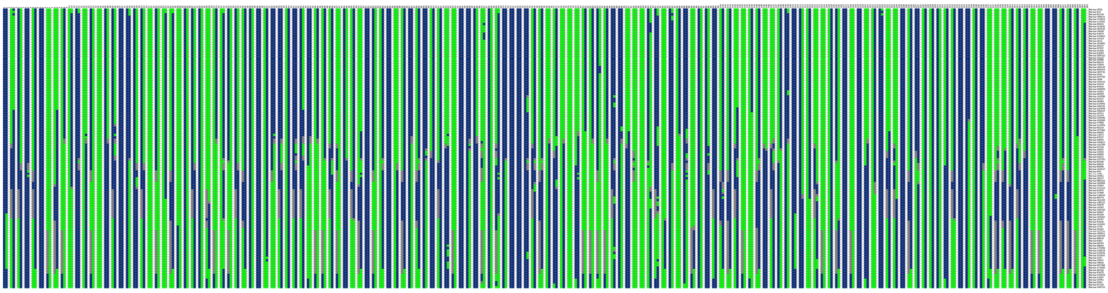

Haplotype map of LG2

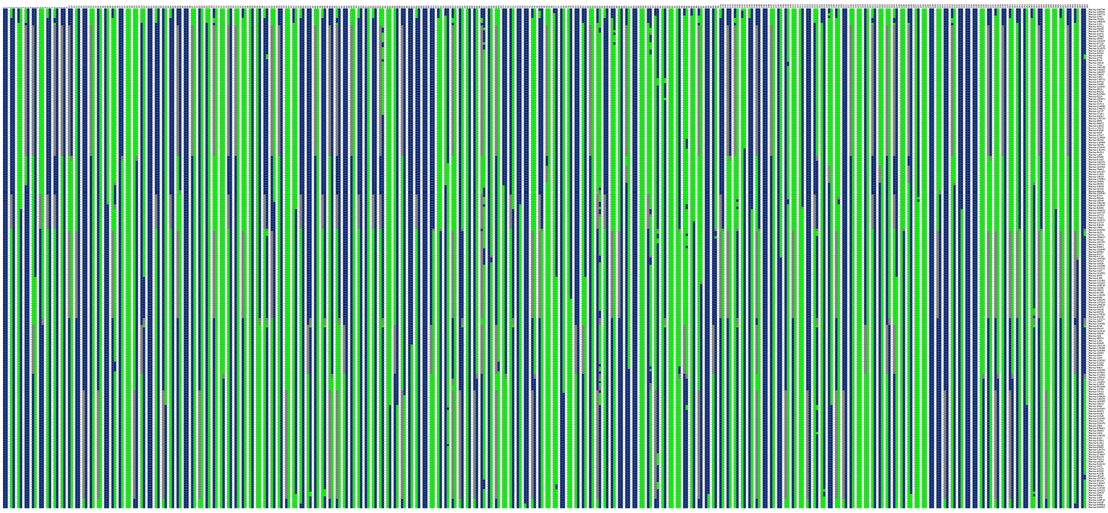

Haplotype map of LG3

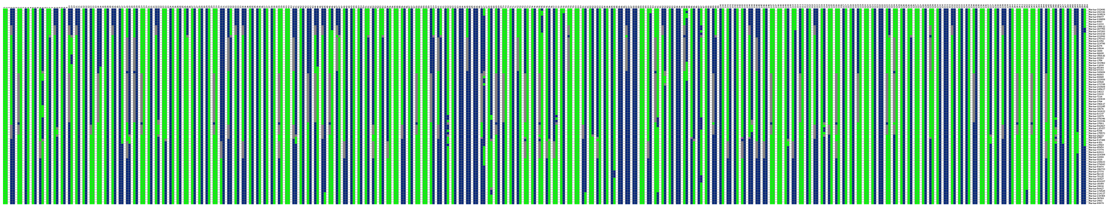

Haplotype map of LG4

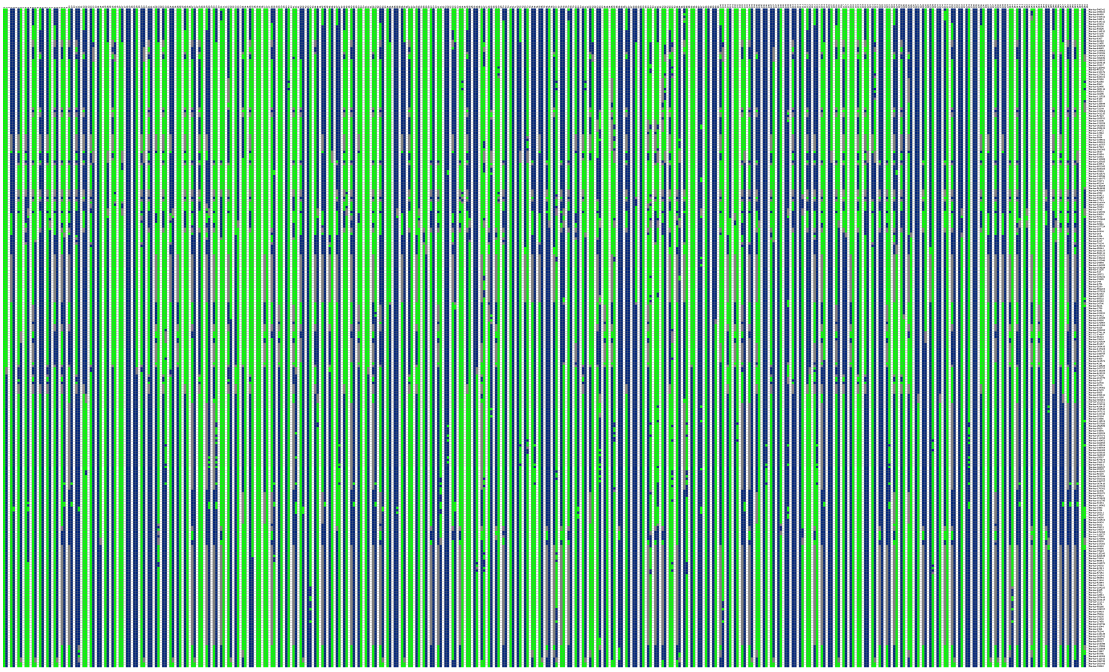

Haplotype map of LG5

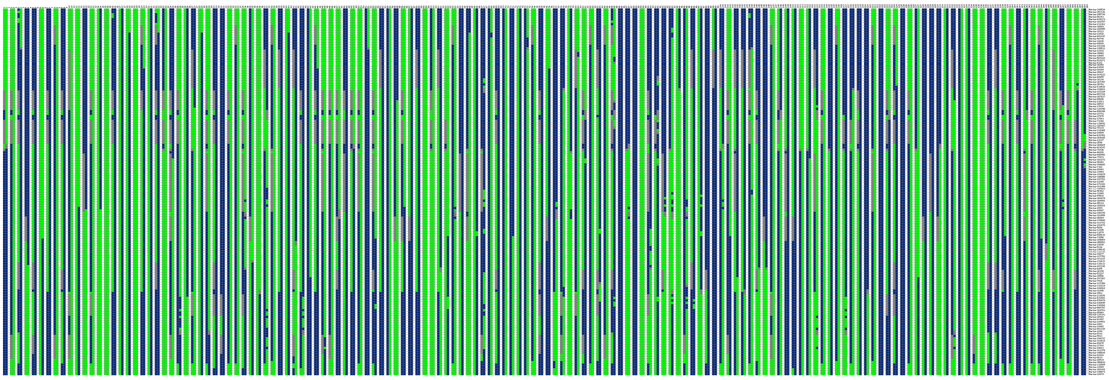

Haplotype map of LG6

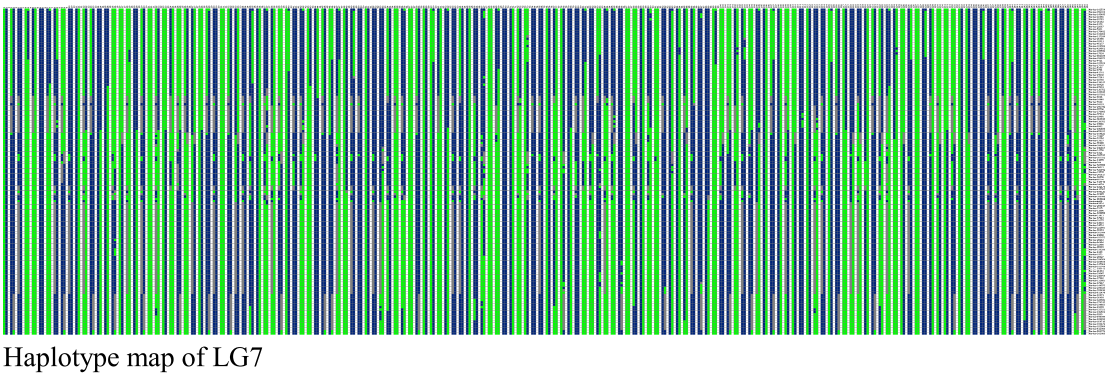

Haplotype map of LG7

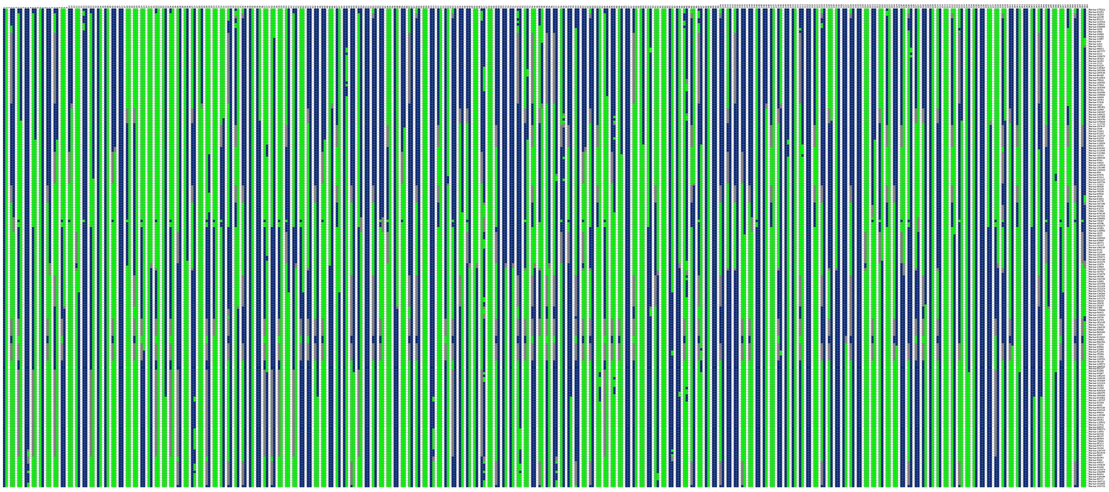

Haplotype map of LG8

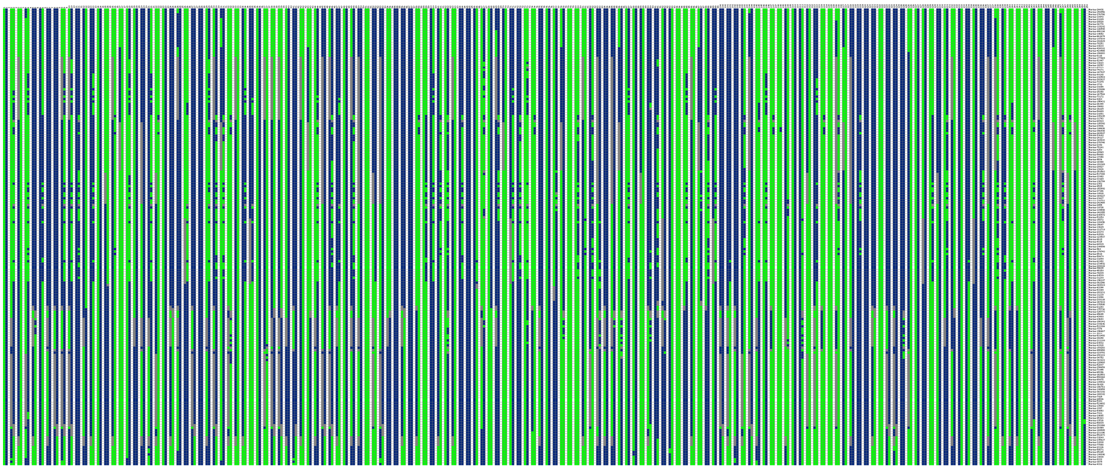

Haplotype map of LG9

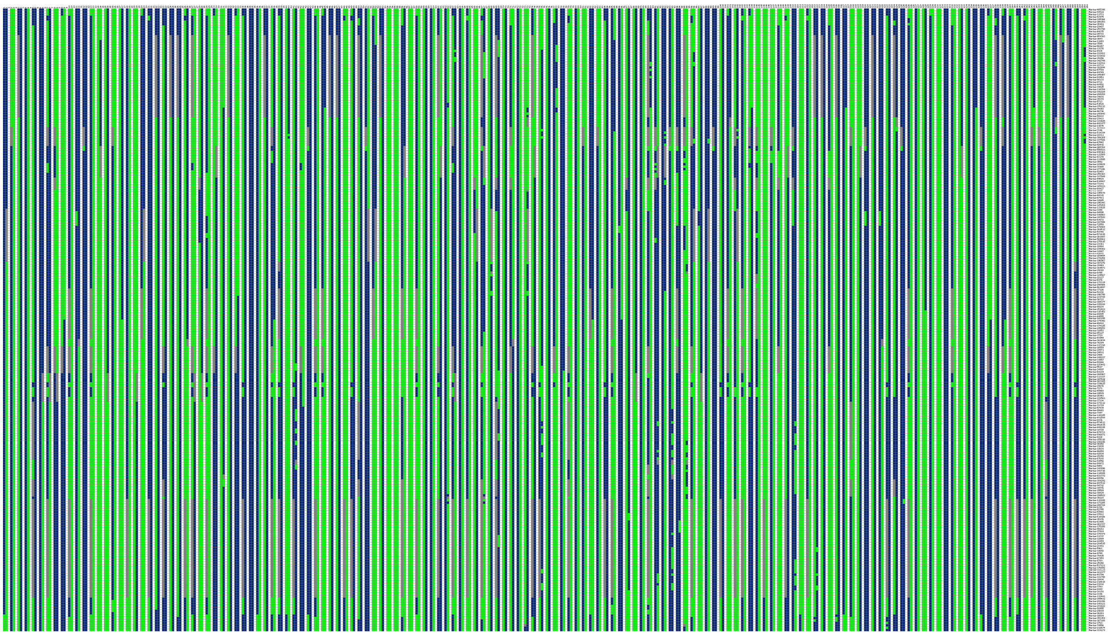

Haplotype map of LG10

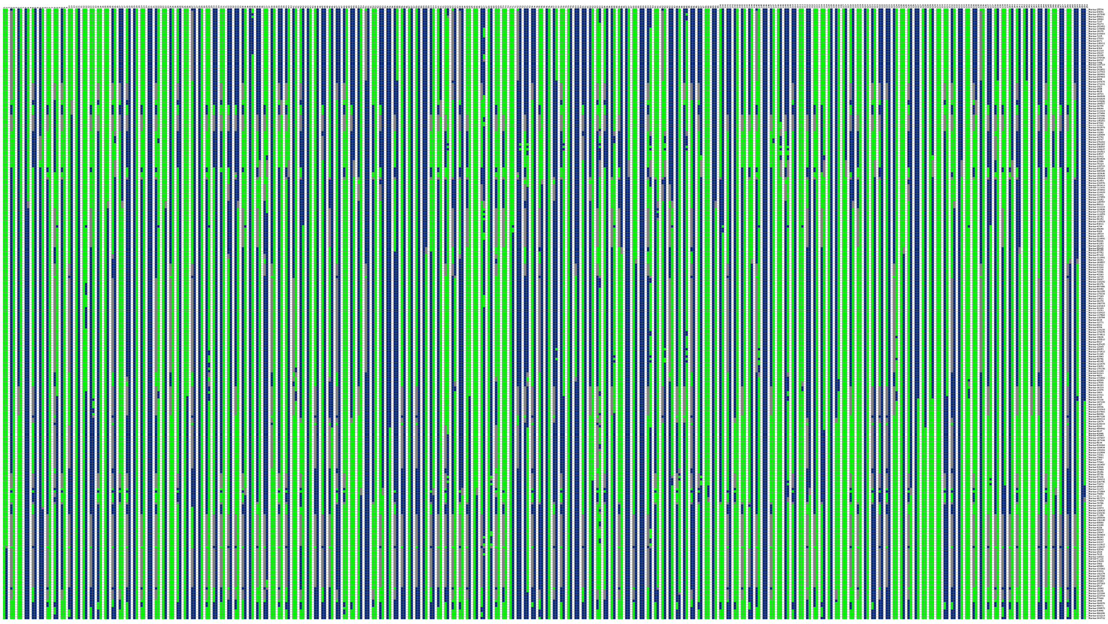

Haplotype map of LG11

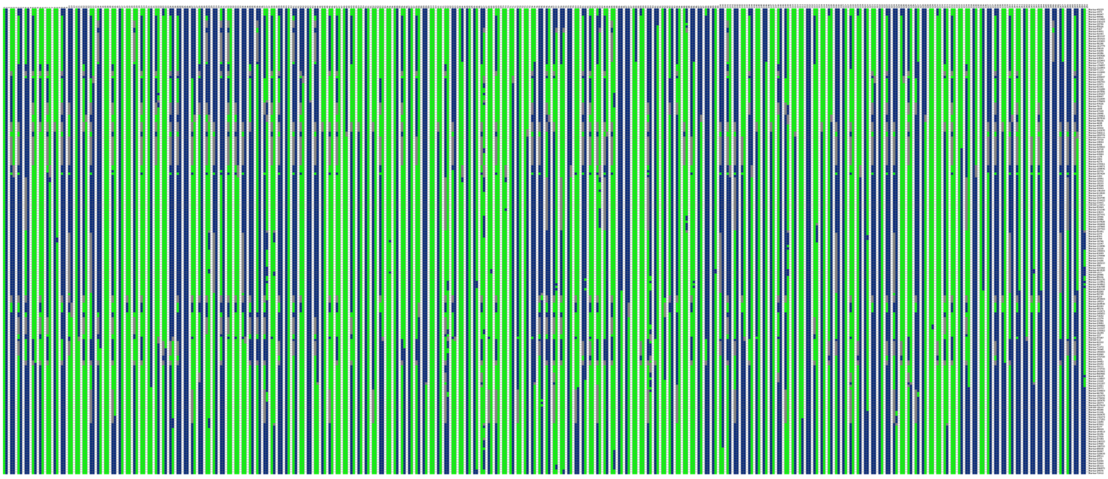

Haplotype map of LG12

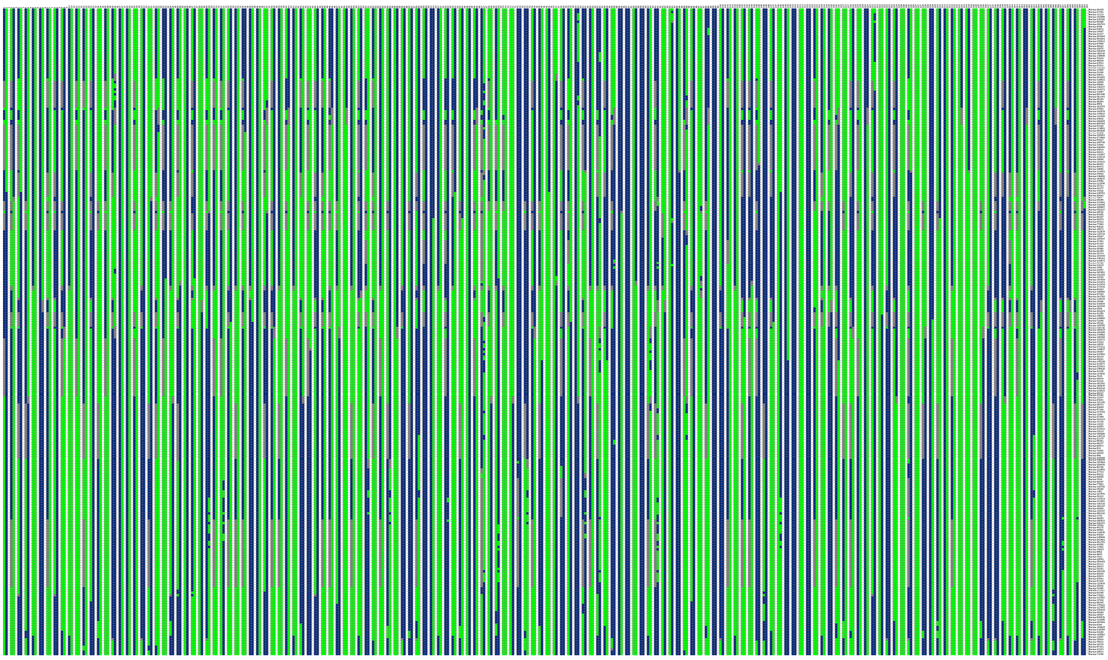

Haplotype map of LG13

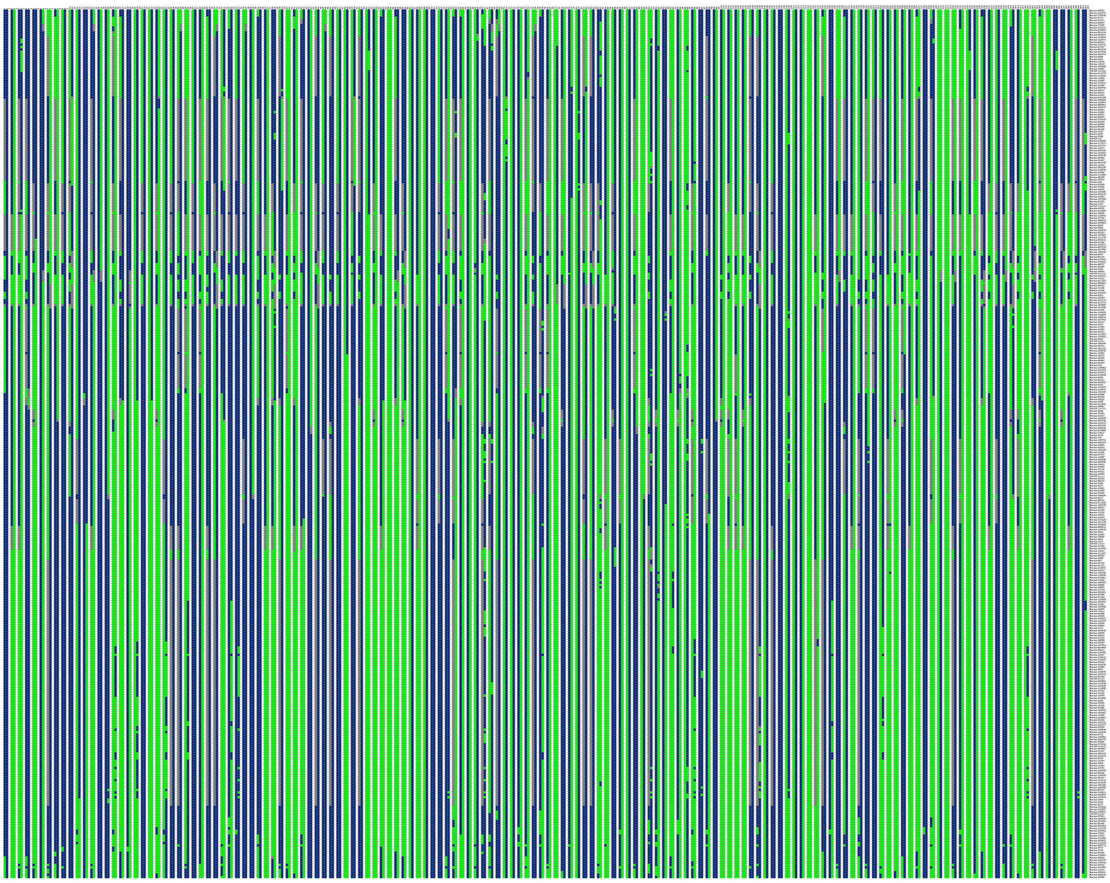

Haplotype map of LG14

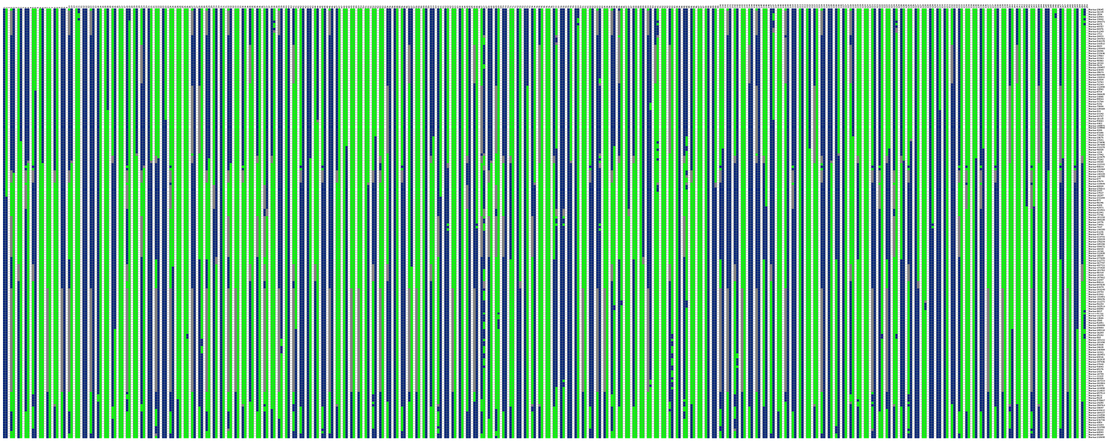

Haplotype map of LG15

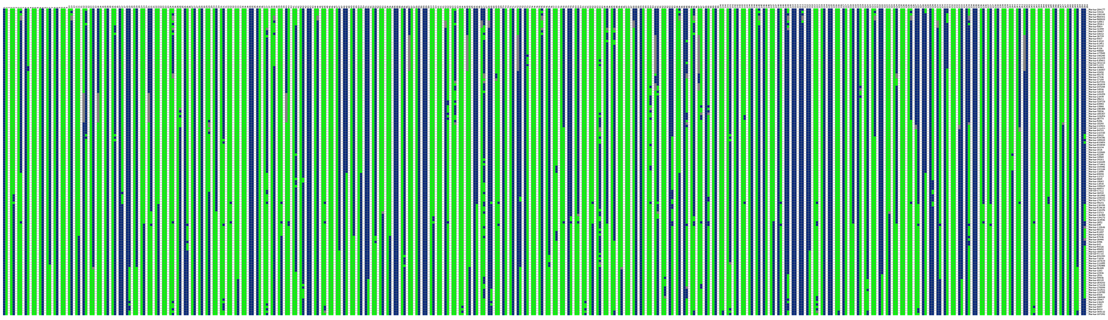

Haplotype map of LG16

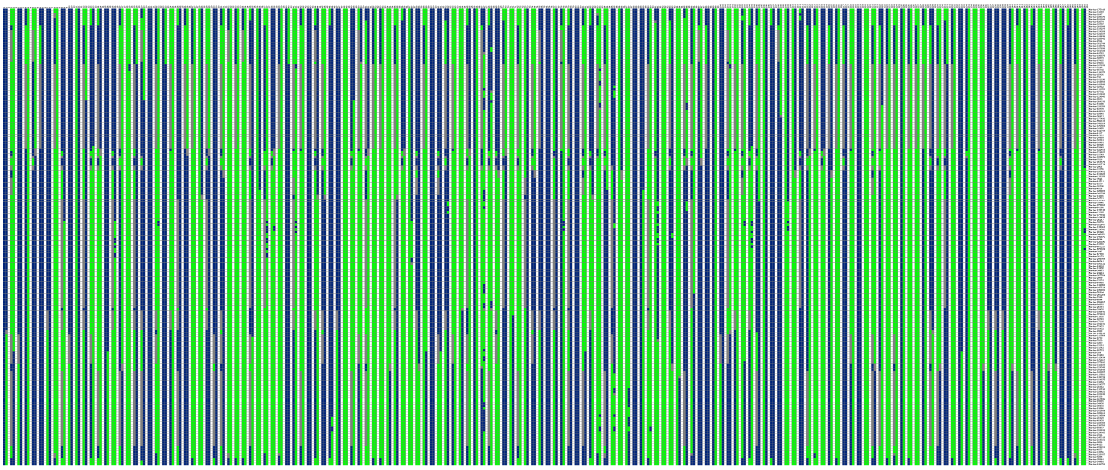

Haplotype map of LG17

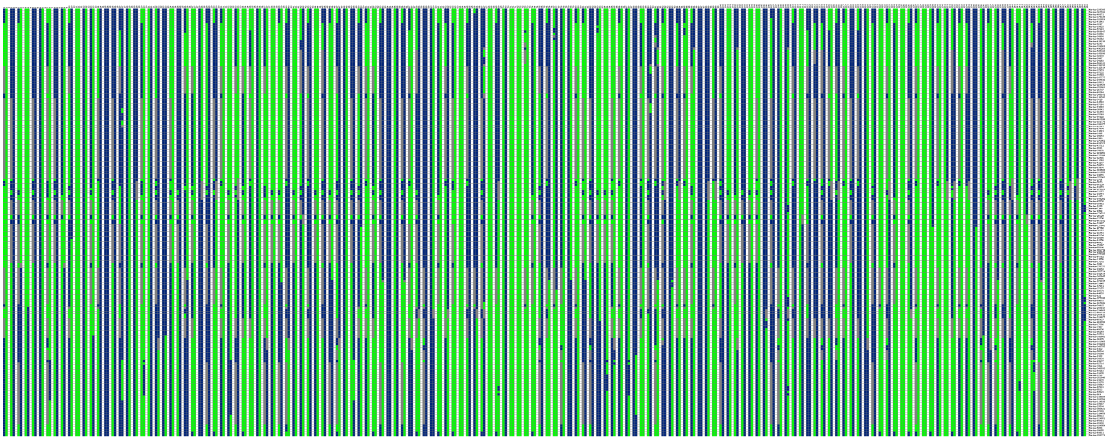

Haplotype map of LG18

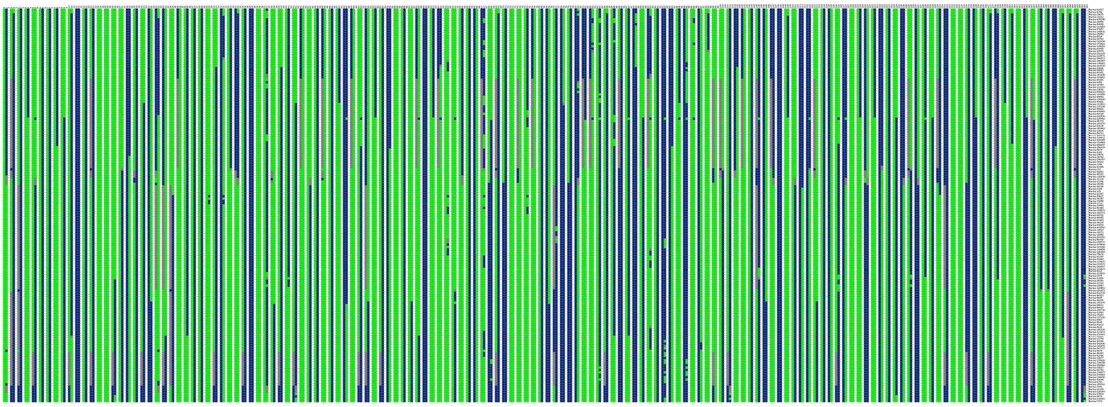

Haplotype map of LG19

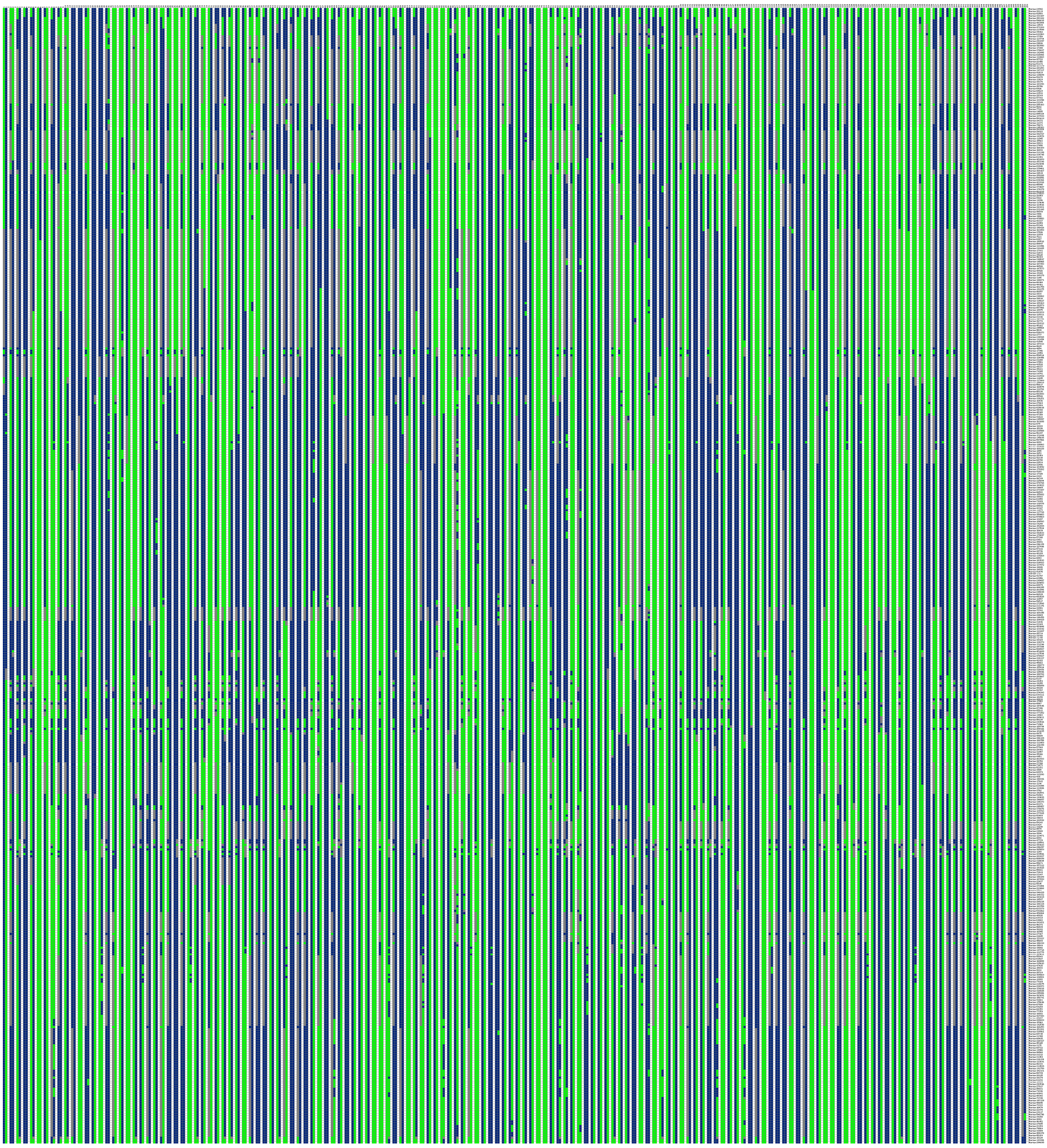

Haplotype map of LG20

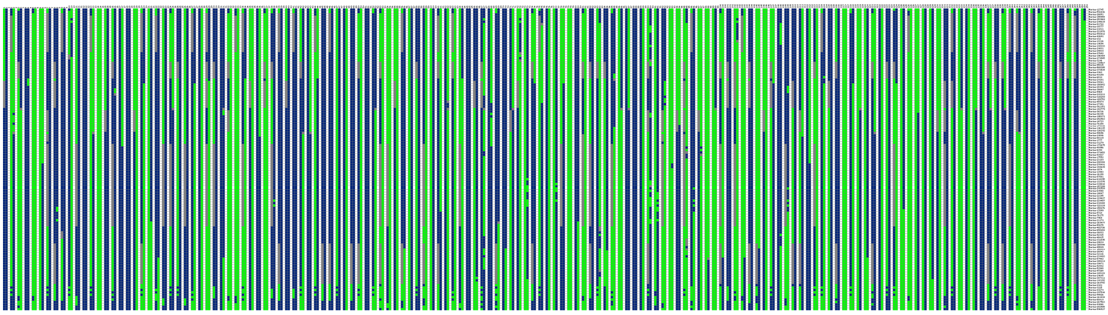

Haplotype map of LG21

Supplementary Figure 5. Haplotype maps of all LGs.
Green represents female parent(2-4), blue represents male parent(15), white means the parent could not be estimated, gray represents deletions.

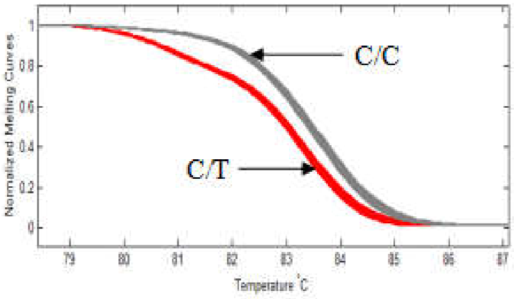

SNP-22（a）

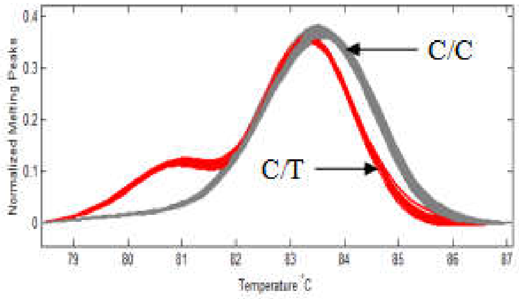

SNP-22（b）

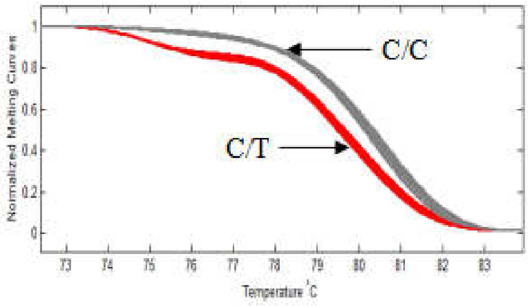

SNP-29（a）

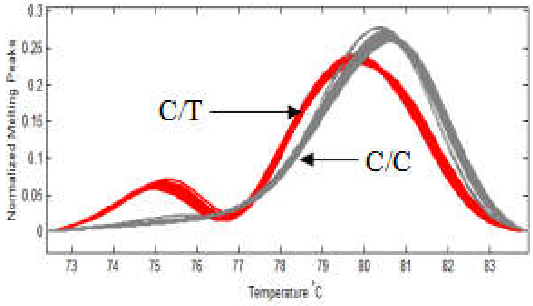

SNP-29（b）

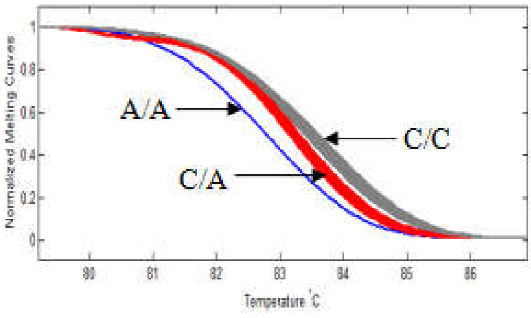

SNP-37（a）

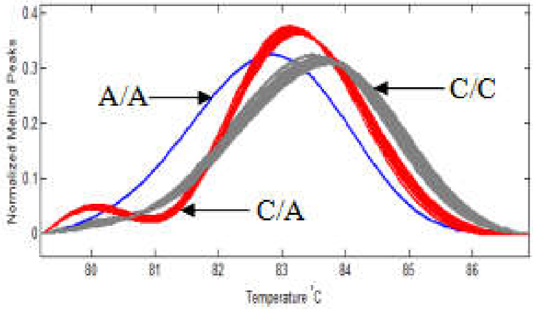

SNP-37（b）

Supplementary Figure 6. HRM genotyping results of the 3 SNPs for verification the SNP markers of the genetic map.

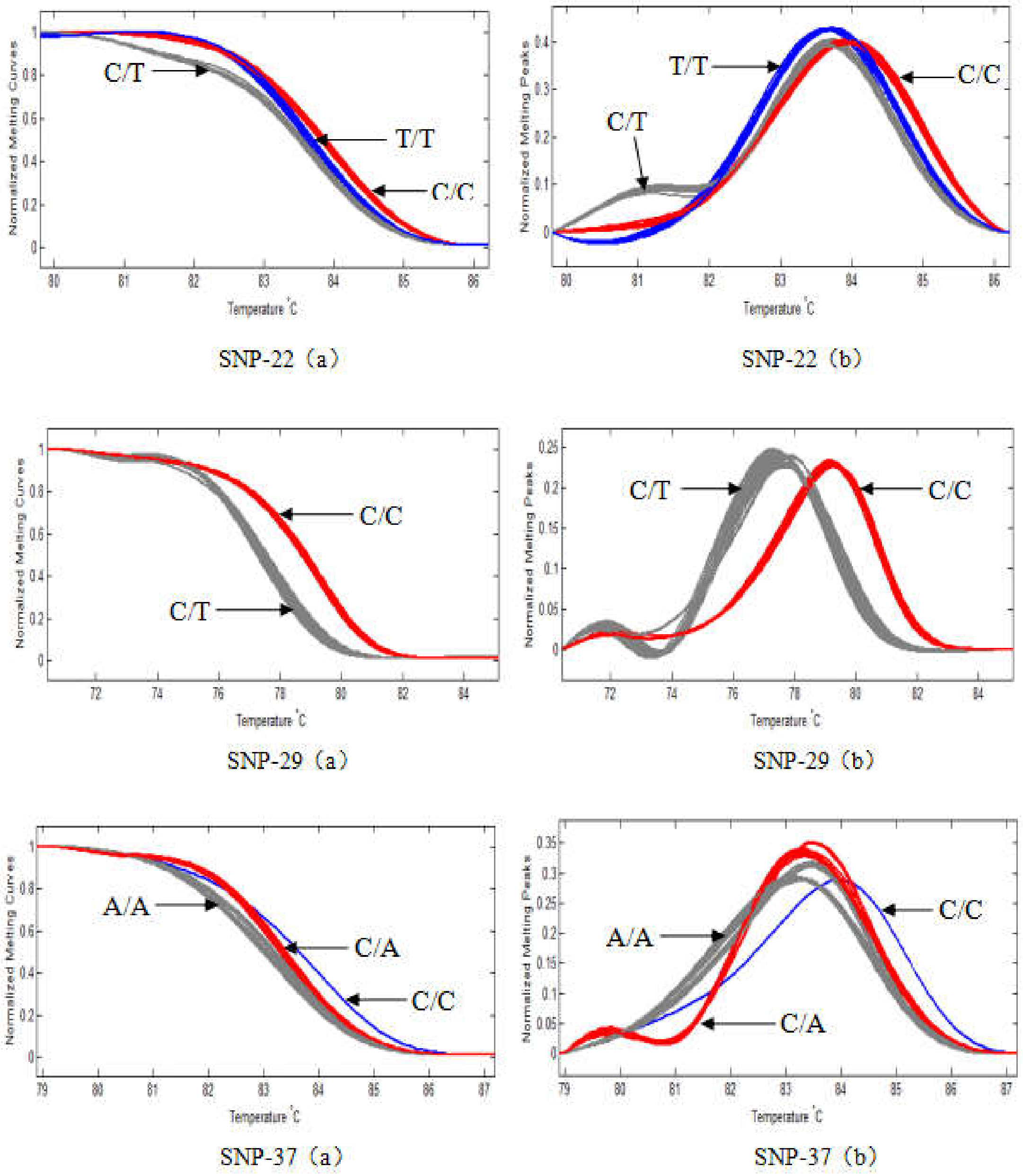

Supplementary Figure 7. HRM genotyping results of three SNP loci in the additional families.
